# KSGR: An Influential Node Identification Algorithm for Directed Networks Integrating Reverse Reachability and Structural Enhancement Mechanisms

**DOI:** 10.3390/e28090981

**Published:** 2026-09-02

**Authors:** Zi-Yue Zhao, Bo-Lin Hu

**Affiliations:** 1 School of Mathematics, Statistics and Mechanics, Beijing University of Technology, Beijing 100124, China; 2 International School, Beijing University of Posts and Telecommunications, Beijing 100876, China; 2023213477@bupt.cn

**Keywords:** directed network, influential node identification, KSGR, reverse diffusion efficiency, SI model

## Abstract

The identification of influential nodes in directed networks is fundamental to diffusion analysis, network robustness assessment, and information recommendation. Owing to the asymmetry introduced by directed edges, conventional centrality methods often struggle to jointly characterize the local spreading range, higher-order diffusion potential, and structural bridging roles. To address this issue, this paper proposes KSGR for influential node identification in directed networks. Under the convention that a node can influence its in-neighbors, KSGR integrates the reverse local reachability, reverse diffusion efficiency, hierarchical asymmetry, directed reverse k-shell, reverse structural gravity, bridging capability, and structural diversity enhancement. A lightweight network-profiling mechanism based on the network size, reciprocity, LWCC ratio, and SCC ratio is further used to select structural enhancement branches for different network profiles. Experiments on six real-world directed networks compare KSGR with PageRank, LeaderRank, ClusterRank, In-degree, Adjacency Entropy, BII, and NEM. Under the adopted reverse-edge propagation convention and SI settings, KSGR achieves the highest average normalized spreading AUC and final infection scale in the Top-10 seed experiments among the selected methods. Supplementary Top-5 and Top-20 experiments further indicate that the ranking can be applied to different seed-set sizes. Kendall correlation analysis shows that KSGR produces rankings distinct from traditional centrality and random-walk-based methods. LWCC node-removal experiments provide evidence that highly ranked KSGR nodes influence the weakly connected backbone of the tested networks. Parameter sensitivity and threshold robustness analyses demonstrate that KSGR maintains stable spreading performance under different enhancement settings and moderate perturbations of adaptive branch-selection thresholds.

## 1. Introduction

Complex networks provide a unified framework for describing individuals and their interactions in real-world systems and have been widely applied in scenarios such as social relationships, communication systems, transportation systems, biological regulation, and information diffusion. In the past five years, related studies have further extended the problem of influential node identification from the perspectives of information diffusion, rumor control, population-level spreading, network inference, and directed higher-order structures [1,2,3,4,5,6,7,8]. Complex systems often involve higher-order synergistic relationships that are difficult to represent using ordinary graphs, and directed hypergraphs and similarity-enhanced ranking have provided new research perspectives for identifying important nodes [9]. A large number of real-world systems are not composed of uniformly connected individuals; instead, they commonly exhibit complex structural characteristics such as heterogeneity, local clustering, and cross-regional connections. In such networks, a small number of nodes occupying special topological positions can often exert a much greater influence than ordinary nodes on overall spreading efficiency, functional organization, and connectivity stability [10,11,12]. Therefore, accurately identifying influential nodes from the network topology is a fundamental problem in complex network analysis, and it also directly supports practical tasks such as public opinion guidance, epidemic immunization, product promotion, network protection, traffic scheduling, and recommender systems.

The problem of influential node identification is also commonly referred to as node centrality evaluation, whose core objective is to assign each node a score that reflects its structural position or spreading potential. Early studies mainly focused on classical centrality measures. Degree centrality characterizes the direct influencing range of a node by using the number of its first-order neighbors, offering the advantages of simple computation and suitability for large-scale networks; however, it cannot distinguish neighbor quality or capture higher-order diffusion potential. Closeness centrality evaluates reachability efficiency based on the average distance from a node to other nodes, whereas betweenness centrality emphasizes a node’s control over the shortest paths [13]. These shortest-path-based metrics are capable of identifying bridging nodes, but they incur high computational costs in large-scale networks and are sensitive to network connectivity and path assumptions. HITS and PageRank utilize link structures for web page ranking and importance evaluations in directed networks [14,15]. More recent studies on the graph-curvature-based PageRank and local-clustering-based H-index further demonstrate that classical ranking ideas can still be combined with local structural features to enhance node discriminability [11,12].

As the network scale increases rapidly, algorithms that rely solely on global paths or random walks have gradually exposed limitations in terms of computational efficiency, convergence conditions, and parameter selection. To achieve a balance between accuracy and efficiency, researchers have proposed many local–global hybrid methods. For example, k-shell decomposition explains differences in node spreading capability by exploiting the core-layer structure of a network [16]; the collective influence method characterizes the global destructive power of influential nodes from the perspective of network dismantling [17]; and influence maximization studies, starting from diffusion models, investigate how a limited number of seed nodes can trigger the maximum spreading range [18]. Meanwhile, recent studies have further enriched the theory and application scenarios of key structural identification from perspectives such as important edge identification, local–global feature fusion, node ranking, and spreading evaluation [10,11,12,19,20,21,22]. These studies indicate that node ranking results must be consistent with the specific spreading mechanism, network directionality, and local coverage redundancy.

Compared with undirected networks, influential node identification in directed networks is more complex. Real-world social platforms, following networks, citation networks, email communication networks, and trust networks often have explicit directions. In real-world directed networks, the edge direction usually encodes asymmetric relationships rather than a universal physical diffusion direction. For example, directed links may represent following relationships in social networks, communication relationships in email systems, citation relationships in academic networks, or trust/dependency relationships in interaction networks. Therefore, the semantic interpretation of the edge direction is application-dependent, and the direction of the observed links does not always directly correspond to the actual direction of information influence. Previous studies have also emphasized that directed networks should be analyzed by considering the specific meaning of asymmetric interactions rather than simply assuming that every edge represents identical spreading behavior [23]. In this work, we adopt a reverse-edge spreading convention to investigate a general structural influence identification problem in directed networks. This assumption is motivated by scenarios where a directed edge i → j indicates that node j can receive or be affected by information originating from node i, such as follower–leader, citation, and dependency-like relationships. Therefore, KSGR does not attempt to infer domain-specific causal influence directions; instead, it provides a direction-consistent structural ranking framework under an explicitly defined propagation convention. For example, in follower–leader networks, edges are usually directed from followers to leaders, while information often spreads from leaders to followers [24,25]. This implies that the outgoing and incoming edges of a node have different roles in terms of spreading semantics: out-neighbors may represent the information sources of the node, whereas in-neighbors may represent the objects that the node can directly influence. Therefore, if directionality is ignored and undirected centrality measures are simply applied, the evaluation of spreading capability may be biased. Recent studies on directed hypergraphs and nonlinear spreading have also shown that directionality, group relationships, and indirect similarity jointly affect the ranking results of important nodes [5,6,7,9].

For directed networks, existing methods have been improved from different perspectives. PageRank characterizes global importance through the stationary distribution of random walks, assuming that nodes pointed to by high-quality nodes are more important [14]. LeaderRank introduces a ground node into the PageRank framework, making the network strongly connected and thereby improving parameter independence and noise resistance [24]. ClusterRank treats the local clustering coefficient as a penalty factor for spreading redundancy, arguing that nodes with lower clustering are more conducive to diffusing information to different local regions [26]. Adjacency Entropy constructs information entropy based on adjacency selection probabilities to characterize uncertainty and structural information in node adjacency relationships [27]. BII balances the local structural information represented by the node in-degree and the global influence accumulated from in-neighbors through an iterative process [28]. NEM further uses the degree-distribution entropy of multi-order in-neighborhoods and out-neighborhoods to describe neighborhood information in directed networks [25]. In addition, ImPageRank incorporates both direct connections and latent similarity relationships into the ranking process by introducing directed hypergraph Jaccard similarity, providing a useful reference for important node identification in directed higher-order networks [9].

Recent studies on influential node identification that are closely related to this paper and place greater emphasis on network structural information mainly focus on local structural fusion, global structural perturbation, geometric structural characterization, and higher-order structural modeling. Among them, local-structure-based methods usually combine clustering coefficients, the H-index, the neighbor degree, or multi-order neighborhood information to characterize the local connection density and neighbor quality around a node. These methods have relatively high computational efficiency, but they often focus more on the node itself and its neighborhood structure, making it difficult to fully describe differences in the spreading direction and coverage redundancy among candidate seeds in directed networks. Structural-entropy-based methods measure the influence of a node on the overall organizational structure by evaluating changes in network structural entropy before and after node removal. They can reflect the role of a node in maintaining network connectivity and structural stability, but their emphasis is generally on global structural perturbation evaluations rather than diffusion capability modeling for specific directed spreading processes. Graph-curvature- or geometric-structure-based methods can identify cross-community connections and bridging nodes, offering good interpretability for network backbones and local curvature characteristics. However, their ranking results still rely heavily on geometric metrics or random-walk frameworks, and they give relatively insufficient consideration to the spreading direction, second-order reverse diffusion, and the complementarity of Top-k seed sets. Methods based on k-shell and entropy fusion further integrate the node shell position, degree information, clustering features, and neighbor weights, enabling the simultaneous use of local information and core backbone structures. Nevertheless, most of these methods are still mainly designed for undirected networks or general complex networks and lack reverse spreading modeling tailored to the semantics of directed edges. By contrast, the KSGR proposed in this paper does not rely on a single structural metric. Under the directed spreading convention that “a node can influence its in-neighbors,” it integrates reverse local reachability, reverse diffusion efficiency, hierarchical asymmetry, directed reverse k-shell, reverse structural gravity, bridging capability, and a structural diversity enhancement mechanism into a unified ranking framework. In this way, KSGR simultaneously characterizes the local diffusion potential of nodes, their positions in the spreading backbone, their cross-regional connection capability, and the coverage complementarity of the candidate node set. Therefore, in terms of structural algorithm design, KSGR places greater emphasis on consistency with the spreading direction, core backbone identification, and redundancy reduction among Top-k seeds, forming a clear distinction from existing structural methods that mainly focus on a single local structure, global perturbation, or geometric feature.

Very recent hybrid approaches further confirm this trend toward combining multiple structural or learning-based signals. For example, Madupuri et al. proposed a hybrid machine-learning and centrality framework for key node identification in complex networks [29], and Tejaswi et al. developed a community-based hybrid centrality algorithm for identifying influential nodes in complex networks [30]. These studies provide useful contemporary baselines and show that influential-node identification is moving from single-index ranking toward feature-fusion and community-aware frameworks. Different from these works, KSGR focuses specifically on directed networks under a reverse-edge spreading convention and explicitly couples reverse reachability, reverse k-shell, structural gravity, and coverage-aware reranking within the same direction-consistent framework.

However, existing methods still leave room for further improvement. First, some algorithms mainly emphasize the number of first-order neighbors or neighbor scores, making it difficult to characterize a node’s ability to expand from its direct influence range to the second-order reverse neighborhood. Second, although random-walk-based and iterative methods can utilize global information, they may be affected by parameters, the number of iterations, and the strongly connected structure of the network. Local entropy methods have lower complexity, but they insufficiently consider the hierarchical position of nodes in the directed spreading backbone and their cross-regional bridging roles. Third, in Top-k seed selection tasks, if multiple high-scoring nodes cover highly overlapping neighborhoods, the overall spreading gain of the seed set may still be limited even when individual node scores are high. Related studies have shown that indirect influence among nodes, latent similarity, and the collaborative coverage of seed sets can significantly affect the final spreading benefit [5,6,9,18,31]. Therefore, an influential node identification algorithm for directed networks should not only focus on the structural strength of individual nodes, but also simultaneously consider the spreading direction, second-order diffusion efficiency, core backbone position, bridging capability, and coverage complementarity among candidate nodes.

Network robustness experiments are also an important way to evaluate influential node identification methods. Changes in the largest connected component after node removal can reflect the supporting role of the identified nodes in the network backbone [10,17]. For directed networks, observing only the Top-k node set makes it difficult to comprehensively judge the influence of ranking results on the overall structure. Gradually deleting high-ranking nodes according to the algorithmic ranking and recording changes in the largest weakly connected component (LWCC) can more intuitively evaluate the ability of different methods to dismantle the network connectivity backbone. Furthermore, a ranking discriminability, monotonicity, and robustness evaluation can supplement spreading capability analysis by indicating whether the node ranking results have stable structural interpretability [9,31]. Therefore, in addition to spreading capability experiments, this paper introduces Kendall correlation analysis and LWCC node-removal experiments to jointly examine the spreading effectiveness, ranking differentiation, and structural influence capability of KSGR.

Based on the above issues, this paper proposes KSGR, an influential node identification algorithm for directed networks. The algorithm is designed for the spreading setting in which “a node can influence its in-neighbors,” and adopts reverse local reachability as the basic spreading direction. On this basis, KSGR constructs reverse diffusion efficiency to measure a node’s ability to expand from its first-order in-neighborhood to its second-order reverse neighborhood, introduces hierarchical asymmetry to characterize the difference between reverse spreading potential and forward outward-expansion structure, and performs directed reverse k-shell decomposition on the reversed graph to obtain shell positions consistent with the spreading direction. Furthermore, KSGR couples the local reachability, shell index, and multi-hop frontier information into reverse structural gravity and reduces coverage redundancy among Top-k candidate nodes through bridging scores and a structural diversity enhancement mechanism. Compared with existing methods, the design objective of KSGR is not merely to improve a single centrality metric, but to unify the directed spreading direction, local diffusion efficiency, structural backbone, and set-level coverage complementarity within the same ranking framework. In particular, KSGR constructs a network profile based on the network scale, reciprocity, proportion of the largest weakly connected component, and proportion of the largest strongly connected component. It then selects the sparse acyclic quota branch, giant strongly connected component entropy-bridging branch, reciprocal-network tail-enhancement branch, or structural diversity enhancement branch accordingly, thereby improving the adaptability of the algorithm across different types of directed networks. Experiments are conducted on six real-world directed networks, and KSGR is compared with seven algorithms: PageRank, LeaderRank, ClusterRank, In-degree, Adjacency Entropy, BII, and NEM [9,14,24,25,26,27,28]. The main contributions of this paper are as follows. First, a reverse diffusion efficiency metric is proposed to measure the ability of a node to expand from its direct in-neighborhood to its second-order reverse neighborhood. Second, hierarchical asymmetry and directed reverse k-shell are introduced so that the node importance evaluation remains consistent with the directed spreading process. Third, reverse structural gravity, bridging capability, and the structural diversity enhancement mechanism are combined to improve the spreading capability of Top-k influential nodes while reducing coverage redundancy among candidate nodes. Fourth, the effectiveness and stability of KSGR are systematically verified on six real-world directed networks through SI spreading experiments, Kendall correlation analysis, LWCC ratio-based node-removal experiments, and parameter sensitivity experiments.

The remainder of this paper is organized as follows. Section 2 introduces the preliminary knowledge and theoretical foundations of the eight comparison algorithms. Section 3 presents the core idea of KSGR, the definitions of its structural features, the adaptive branch selection mechanism, and the theoretical motivation for the proposed feature fusion. Section 4 describes the SI spreading model, the LWCC node-removal experiment, and the ranking correlation evaluation metrics. Section 5 reports the experimental results on six real-world directed networks, analyzes the performance differences among different algorithms, and discusses computational complexity. Section 6 concludes the paper, and Section 7 discusses future research directions.

## 2. Preliminary Knowledge and Theoretical Foundations of Comparison Algorithms

Let the directed network be denoted as G=(V,E), where V is the set of nodes and E is the set of directed edges. If the directed edge $\left(v_{i},v_{j}\right)\in E$, then $a_{ij}=1$; otherwise, $a_{ij}=0$. The in-neighbor set and out-neighbor set of node $v_{i}$ are denoted respectively as follows:

$$\Gamma^{-}\left(v_{i}\right)=v_{j}\left|\left(v_{j},v_{i}\right)\in E,\Gamma^{+}\left(v_{i}\right)=v_{j}\right|\left(v_{i},v_{j}\right)\in E.$$

Under the spreading convention adopted in this paper, if node A points to node B, then node B can influence node A along the reverse edge; that is, spreading proceeds in the direction of predecessors. To highlight the theoretical foundations of different algorithms, this paper compares eight ranking methods: PageRank, LeaderRank, ClusterRank, In-degree, Adjacency Entropy, BII, NEM, and KSGR [9,14,24,25,26,27,28].

Influential node identification methods have been developed from different perspectives, including local structural characteristics, global ranking mechanisms, entropy-based information measures, and higher-order neighborhood aggregation. Since KSGR is proposed as a topology-based influential node identification algorithm for directed networks, this paper selects representative topology-driven algorithms as comparison methods rather than learning-based prediction models that require additional node attributes or training information. The selected algorithms cover several important methodological categories: PageRank and LeaderRank represent random-walk-based ranking approaches; In-degree and ClusterRank characterize local structural importance; Adjacency Entropy introduces information-theoretic measurements; BII integrates local and global structural influence; and NEM considers higher-order neighborhood entropy information in directed networks. Therefore, the comparison set provides a comprehensive evaluation of KSGR from multiple structural perspectives, as summarized in Table 1.

### 2.1. PageRank

PageRank was originally proposed for ranking web pages [14]. Its basic idea is that the importance of a node depends not only on the number of nodes pointing to it, but also on the importance of those pointing nodes themselves. Let c denote the damping factor and N the number of nodes. The iterative form of PageRank can be written as follows:

$$PR\left(v_{i}\right)=\frac{1-c}{N}+c\sum_{v_{j}\in \Gamma^{-}\left(v_{i}\right)}\frac{PR\left(v_{j}\right)}{d_{out}\left(v_{j}\right)}$$

where $PR\left(v_{i}\right)$ denotes the PageRank score of node $v_{i}$; c is the damping factor; N is the total number of nodes in the network; $\Gamma^{-}\left(v_{i}\right)$ is the in-neighbor set of node $v_{i}$; and $d_{out}\left(v_{j}\right)$ is the out-degree of node $v_{j}$.

### 2.2. LeaderRank

LeaderRank inherits the random-walk framework of PageRank [24], but extends the original network into a strongly connected network by introducing a ground node that is bidirectionally connected to all real nodes. Each real node is assigned an initial resource of 1, while the initial resource of the ground node is set to 0. Node resources are then evenly distributed along outgoing edges until a steady state is reached.

(1) $$s_{i}\left(t+1\right)=\sum_{v_{j}\in \Gamma^{-}\left(v_{i}\right)}a_{ji}\frac{s_{j}\left(t\right)}{d_{out}\left(v_{j}\right)}.$$

where ${\;s}_{i}\left(t+1\right)$ denotes the resource obtained by node $v_{i}$ at step t+1; $a_{ji}$ indicates whether node $v_{j}$ points to node $v_{i}$; $d_{out}\left(v_{j}\right)$ is the out-degree of node $v_{j}$; and $s_{j}\left(t\right)$ denotes the resource of node $v_{j}$ at step t.

(2) $$S_{i}=s_{i}\left(t_{c}\right)+\frac{s_{g}\left(t_{c}\right)}{N}.$$

where $S_{i}$ denotes the final LeaderRank score of node $v_{i}$; $s_{i}\left(t_{c}\right)$ is the resource value of node $v_{i}$ at convergence; $s_{g}\left(t_{c}\right)$ is the steady-state resource value of the ground node; and N is the number of real nodes.

### 2.3. ClusterRank

ClusterRank emphasizes the negative effect of local clustering on spreading [26]. If the neighbors of a node are highly clustered, information is likely to spread repeatedly within a local cluster; conversely, nodes with lower clustering are more likely to connect different regions.

(3) $$CR\left(v_{i}\right)=f\left(c_{i}\right)\sum_{v_{j}\in \Gamma^{-}\left(v_{i}\right)}\left(d_{in}\left(v_{j}\right)+1\right),f\left(c_{i}\right)=2^{-c_{i}}.$$

### 2.4. In-Degree

In-degree is one of the most basic indicators for evaluating node importance in directed networks. The in-degree of node $v_{i}$ represents the number of edges pointing to that node. In follower–leader-type directed networks, edges are typically directed from followers to leaders, while the direction of information diffusion is opposite to the edge direction.

(4) $$ID\left(v_{i}\right)=d_{in}\left(v_{i}\right)=\sum_{j}a_{ji}.$$

where $ID\left(v_{i}\right)$ denotes the in-degree score of node $v_{i}$; $d_{in}\left(v_{i}\right)$ is the in-degree of node $v_{i}$; and $a_{ji}$ indicates whether node $v_{j}$ points to node $v_{i}$.

### 2.5. Adjacency Entropy

Adjacency Entropy uses the network adjacency matrix to define node adjacency information entropy for identifying important nodes [27]. This method introduces the parameter θ to balance the contributions of in-degree and out-degree. A larger entropy value indicates greater uncertainty in adjacency selection and richer structural information.

Specifically, the balanced degree, neighborhood degree, and adjacency selection probability are defined respectively as follows:

$$k_{\theta }\left(v_{i}\right)=\theta d_{in}\left(v_{i}\right)+\left(1-\theta \right)d_{out}\left(v_{i}\right)$$

where ${\;k}_{\theta }\left(v_{i}\right)$ denotes the in–out degree balanced degree of node $v_{i}$; θ is the in-degree weighting parameter; and $d_{in}\left(v_{i}\right)$ and $d_{out}\left(v_{i}\right)$ denote the in-degree and out-degree of node $v_{i}$, respectively.

$$A_{\theta }\left(v_{j}\right)=\theta \sum_{u\in \Gamma^{+}\left(v_{j}\right)}d_{in}\left(u\right)+\left(1-\theta \right)\sum_{u\in \Gamma^{+}\left(v_{j}\right)}d_{out}\left(u\right)$$

where $A_{\theta }\left(v_{j}\right)$ denotes the adjacency degree of node $v_{j}$; $\Gamma^{+}\left(v_{j}\right)$ is the out-neighbor set of node $v_{j}$; and $d_{in}\left(u\right)$ and $d_{out}\left(u\right)$ denote the in-degree and out-degree of the neighboring node u, respectively.

$$P_{ij}=\frac{k_{\theta }\left(v_{i}\right)}{A_{\theta }\left(v_{j}\right)}$$

where $P_{ij}$ denotes the probability that neighboring node $v_{j}$ selects the target node $v_{i}$ during the spreading process; $k_{\theta }\left(v_{i}\right)$ is the balanced degree of node $v_{i}$; and $A_{\theta }\left(v_{j}\right)$ is the adjacency degree of node $\;v_{j}$.

Therefore, the Adjacency Entropy of node $v_{i}$ is defined as follows:

(5) $$E_{i}=\sum_{v_{j}\in \Gamma^{+}\left(v_{i}\right)}\left|-P_{ij}\log_{2}P_{ij}\right|.$$

where $E_{i}$ denotes the adjacency information entropy of node $v_{i}$; $\Gamma^{+}\left(v_{i}\right)$ is the neighbor set of node $v_{i}$; and $P_{ij}$ is the adjacency selection probability.

### 2.6. Balanced Iterative Influence (BII)

BII is an iterative influence algorithm designed for directed networks [28]. It assumes that the local structural information of a node and the accumulated influence from its in-neighbors have different physical meanings and that the two should be balanced during the iterative process.

(6) $$x_{i}^{\left(t+1\right)}=\alpha \sum_{v_{j}\in \Gamma^{-}\left(v_{i}\right)}x_{j}^{\left(t\right)}+d_{in}\left(v_{i}\right).$$

where $x_{i}^{\left(t+1\right)}$ denotes the influence value of node $v_{i}$ at step t+1; α is the global influence adjustment parameter; $\Gamma^{-}\left(v_{i}\right)$ is the in-neighbor set of node $v_{i}$; $x_{j}^{\left(t\right)}$ denotes the influence value of the in-neighbor $v_{j}$ at step  t; and $d_{in}\left(v_{i}\right)$ represents the local in-degree information of node $v_{i}$.

(7) $$x_{i}^{\left(t+1\right)}\leftarrow \frac{x_{i}^{\left(t+1\right)}}{\sqrt{\sum_{v_{l}\in V}{\left(x_{l}^{\left(t+1\right)}\right)}^{2}}}.$$

where this equation denotes the L2 normalization of the BII iterative influence vector; V is the node set; and $x_{l}^{\left(t+1\right)}$ is the influence value of node $v_{l}$ at step t+1.

### 2.7. NEM

NEM is an influential node identification algorithm for directed networks based on neighborhood entropy [25]. It assumes that node influence depends not only on the number of neighbors, but also on the degree distributions of multi-order in-neighbors and out-neighbors, and uses a decay factor to regulate the contributions of higher-order neighbors.

(8) $$NIA\left(v_{i}\right)=\alpha E_{in}\left(v_{i}\right)+\left(1-\alpha \right)E_{out}\left(v_{i}\right).$$

where $NIA\left(v_{i}\right)$ denotes the neighbor influence ability of node $v_{i}$; $E_{in}\left(v_{i}\right)$ and $E_{out}\left(v_{i}\right)$ denote the in-neighborhood entropy and out-neighborhood entropy of node $v_{i}$, respectively; and α is used to balance the contributions of in-neighbors and out-neighbors.

NEM incorporates higher-order neighborhood entropy information and considers multi-order structural characteristics in directed networks. Therefore, it is selected as a representative advanced topology-based baseline.

### 2.8. Collective Influence-Related Discussion

In addition to centrality-based and neighborhood-information-based approaches, collective influence has emerged as an important perspective for identifying influential nodes by considering the contribution of nodes to large-scale network connectivity and influence propagation. Such methods have been widely studied in applications including network immunization, node removal, and influence maximization. However, many existing collective influence-based approaches are mainly developed for undirected networks and focus on identifying nodes whose removal or activation can significantly affect the global network structure. Different from these methods, KSGR incorporates the collective influence perspective into directed influential node identification under a direction-consistent spreading assumption. Specifically, KSGR considers the complementary contribution among multiple candidate nodes through reverse two-hop coverage and introduces a structural diversity enhancement mechanism to reduce redundant influence coverage during Top-k seed selection. Therefore, KSGR extends the idea of collective influence from global connectivity-oriented analysis toward directed spreading-oriented node ranking by jointly considering reverse reachability, the structural backbone position, and coverage complementarity.

## 3. KSGR Algorithm

**Figure 1:**
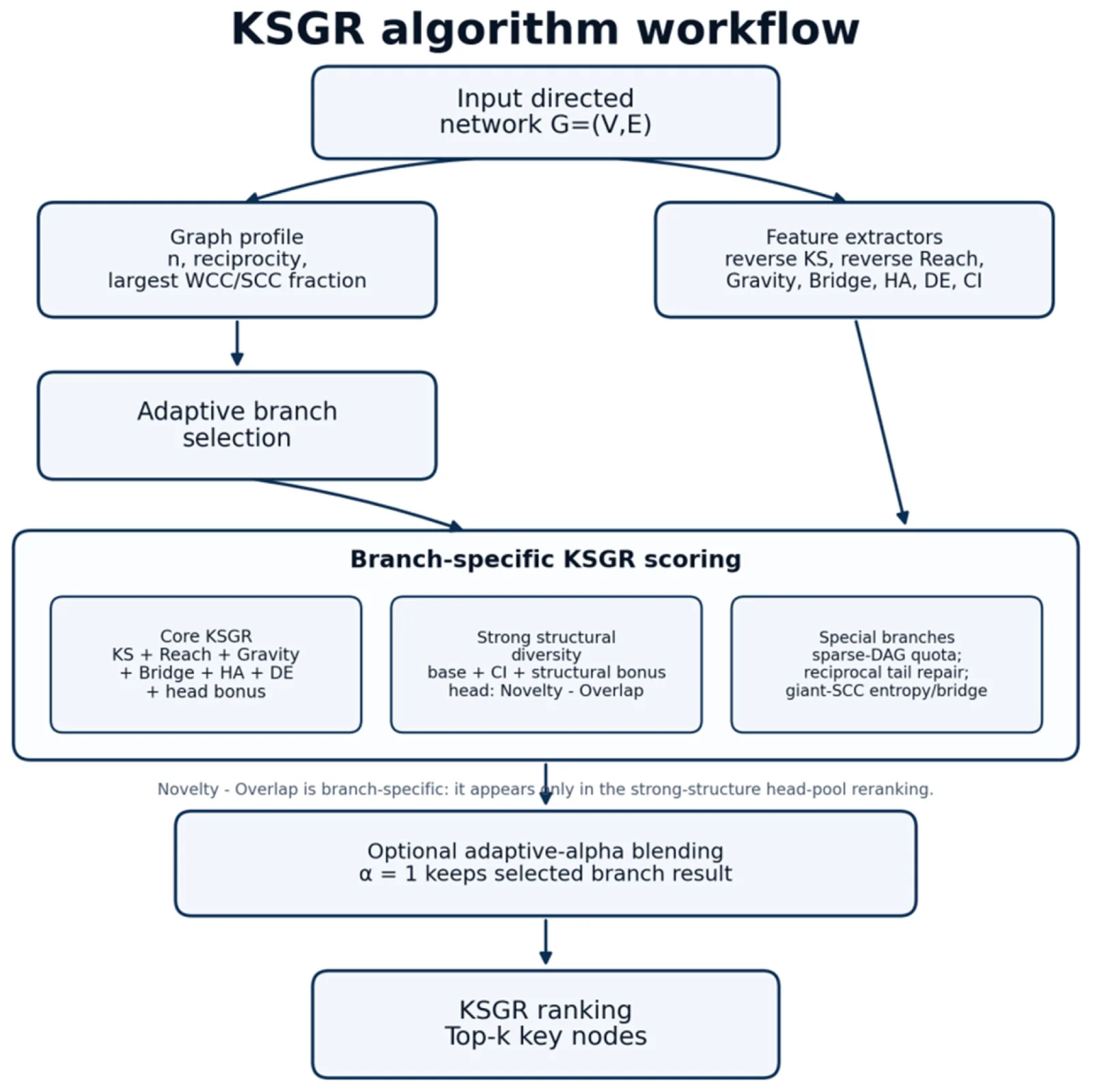
Schematic illustration of the overall workflow of the KSGR algorithm. This figure presents the main computational procedure of KSGR. First, a directed network is taken as input, and node in-neighborhoods, out-neighborhoods, and network-profile information, including the network size, reciprocity, proportion of the largest weakly connected component, and proportion of the largest strongly connected component, are extracted. Then, reverse local reachability, reverse diffusion efficiency, hierarchical asymmetry, directed reverse k-shell, reverse structural gravity, bridging capability, and the structural diversity enhancement term are calculated. Finally, node importance ranking results are obtained through adaptive branch selection and comprehensive scoring.

The core idea of KSGR is that, in directed spreading scenarios, influential nodes should not only possess a large direct in-neighborhood, but also have the ability to further expand from the first-order in-neighborhood to the second-order reverse neighborhood, while occupying positions in the directed structural backbone and cross-regional bridging locations. To this end, KSGR constructs a set of direction-consistent structural features and adapts to different network types through adaptive branch selection. The overall workflow of KSGR is illustrated in Figure 1.

### 3.1. Reverse Local Reachability and Reverse Diffusion Efficiency

Let $\Gamma^{-}\left(v\right)$ denote the in-neighbor set of node v. The reverse second-order neighborhood is defined as follows:

(9) $$R_{2}\left(v\right)=\left(\underset{u\in \Gamma^{-}\left(v\right)}{⋃}\Gamma^{-}\left(u\right)\right)\setminus \Gamma^{-}\left(v\right)\setminus \{v\}.$$

where $R_{2}\left(v\right)$ denotes the second-order reverse neighborhood of node v; $\Gamma^{-}\left(v\right)$ is the in-neighbor set of node v; and $\Gamma^{-}\left(u\right)$ is the in-neighbor set of the in-neighbor u. The set difference operation is used to exclude the first-order in-neighbors and node v itself. Specifically, the first-order in-neighbors of node v and node v itself are removed from the union of the in-neighborhoods of its first-order in-neighbors, thereby obtaining the true second-order reverse neighborhood.

Reverse local reachability characterizes the first-order and second-order coverage range that a node can reach along the spreading direction, where β is the weight assigned to the second-order reverse neighborhood:

(10) $$LR^{-}\left(v\right)=\left|\Gamma^{-}\left(v\right)\right|+\beta \left|R_{2}\left(v\right)\right|.$$

where $LR^{-}\left(v\right)$ denotes the reverse local reachability of node v; $\left|\Gamma^{-}\left(v\right)\right|$ is the number of first-order in-neighbors; $\left|R_{2}\left(v\right)\right|$ is the size of the second-order reverse neighborhood; and *β* is the contribution weight of the second-order neighborhood.

KSGR further proposes reverse diffusion efficiency, denoted as DE, to measure the efficiency with which a node expands from its direct in-neighborhood to the second-order reverse neighborhood:

(11) $$DE\left(v\right)=\log\left(1+\frac{\left|R_{2}\left(v\right)\right|}{\left|\Gamma^{-}\left(v\right)\right|+1}\right).$$

where DE(v) denotes the reverse diffusion efficiency of node v; $\left|R_{2}\left(v\right)\right|$ represents the size of the second-order reverse neighborhood; $\left|\Gamma^{-}\left(v\right)\right|$ represents the size of the first-order in-neighborhood; and the logarithmic function is used to suppress excessive amplification caused by extremely large neighborhoods.

### 3.2. Hierarchical Asymmetry

In directed networks, the reverse spreading potential and forward outward-expansion structure are not equivalent. To distinguish nodes that primarily serve as spreading sources from ordinary connecting nodes, KSGR defines the forward local reachability as $LR^{+}\left(v\right)=\left|\Gamma^{+}\left(v\right)\right|+\beta \left|\left({⋃}_{u\in \Gamma^{+}\left(v\right)}\Gamma^{+}\left(u\right)\right)\setminus \Gamma^{+}\left(v\right)\setminus \{v\}\right|$ and constructs hierarchical asymmetry as follows:

(12) $$HA\left(v\right)=\log\left(1+\frac{LR^{-}\left(v\right)+1}{LR^{+}\left(v\right)+1}\right).$$

where HA(v) denotes hierarchical asymmetry; $LR^{-}\left(v\right)$ and $LR^{+}\left(v\right)$ represent the reverse local reachability and forward local reachability of node v, respectively. This metric is used to measure the advantage of the reverse spreading potential over the forward outward-expansion structure.

### 3.3. Directed Reverse k-Shell and Structural Gravity

KSGR performs k-shell decomposition on the reversed graph GR [16] to obtain a shell index that is consistent with the reverse spreading direction. Since the out-degree in GR corresponds to in-neighborhood expansion in the original graph, the reverse k-shell can reflect the core position of a node in the spreading backbone.

(13) $$M\left(v\right)=\left(LR^{-}\left(v\right)+1\right)\left(KS\left(v\right)+1\right).$$

where M(v) denotes the structural mass of node v; $LR^{-}\left(v\right)$ denotes reverse local reachability; KS(v) is the k-shell index of node v in the reversed graph; and “+1” is used to prevent nodes with zero values from losing their contribution.

(14) d(v,u)=h+|KS(v)−KS(u)|.

where d(v,u) denotes the structural distance between node v and the reverse frontier node u; h is the number of reverse hops; and KS(v) and KS(u) denote the shell indices of nodes v and u, respectively.

(15) $$G\left(v\right)=\sum_{h=1}^{H}\sum_{u\in F_{h}^{-}\left(v\right)}\rho^{h-1}\frac{M\left(v\right)M\left(u\right)}{d{\left(v,u\right)}^{2}+\varepsilon }.$$

where G(v) denotes the reverse structural gravity of node v; $F_{h}^{-}\left(v\right)$ is the set of h-order reverse frontier nodes; H is the maximum search depth; ρ is the hop decay coefficient; M(v) and M(u) denote the masses of nodes v and u, respectively; and ε is used to avoid division by zero.

### 3.4. Bridging Capability and Structural Diversity Enhancement

To capture a node’s cross-community spreading capability, KSGR defines the bridging score on the undirected projection GU as follows:

(16) $$B\left(v\right)=\left(1-C_{U}\left(v\right)\right)\log\left(1+k_{U}\left(v\right)\right).$$

where B(v) denotes the bridging score of node  v; $C_{U}\left(v\right)$ is the clustering coefficient of node v on the undirected projection $\hat{GU}$; and $k_{U}\left(v\right)$ is the undirected degree. Nodes with low clustering and high connectivity have stronger bridging capability.

In the structural diversity enhancement branch, KSGR further draws on the idea of collective influence [17] and performs greedy reranking on the Top candidate pool. Let F(v) denote the reverse two-hop coverage footprint of node v, and let C be the union of the coverage footprints of the already selected nodes. Then,

(17) $$Novelty\left(v\right)=\frac{\left|F\left(v\right)\setminus C\right|}{\left|F\left(v\right)\right|},Overlap\left(v\right)=\frac{\left|F\left(v\right)\cap C\right|}{\left|F\left(v\right)\cup C\right|}.$$

where Novelty(v) denotes the proportion of additional coverage contributed by candidate node v relative to the already selected set; Overlap(v) denotes the degree of overlap between the coverage footprint of candidate node v and the coverage footprints of the already selected nodes; F(v) is the reverse two-hop coverage footprint of node v; and C is the union of the coverage footprints of the already selected nodes.

(18) $$Q\left(v\right)=S_{boost}\left(v\right)+\eta_{h}H\left(v\right)+\eta_{s}T\left(v\right)+\eta_{n}Novelty\left(v\right)-\eta_{o}Overlap\left(v\right).$$

where Novelty(v) measures the proportion of additional coverage introduced by the candidate node, and Overlap(v) measures the redundancy between the coverage range of the candidate node and that of the selected nodes; $\eta_{h}$, $\eta_{s}$, $\eta_{n}$, and $\eta_{o}$ are the weighting coefficients of the corresponding terms in structural diversity reranking.

### 3.5. Core Scoring and Adaptive Branch Selection

For any structural indicator x(v), this paper applies min–max normalization:

$$\hat{x}\left(v\right)=\frac{x\left(v\right)-\underset{u\in V}{\min\;}x\left(u\right)}{\underset{u\in V}{\max\;}x\left(u\right)-\underset{u\in V}{\min\;}x\left(u\right)}.$$

where $\hat{x}\left(v\right)$ denotes the min–max normalized value of indicator x on node v; min and max represent the minimum and maximum values of this indicator over all nodes, respectively.

KSGR normalizes and weights the reverse k-shell, reverse reachability, reverse structural gravity, bridging capability, hierarchical asymmetry, and reverse diffusion efficiency to form the core score:

(19) $$S_{base}\left(v\right)=w_{ks}\hat{KS}\left(v\right)+w_{r}\hat{LR^{-}}\left(v\right)+w_{g}\hat{G}\left(v\right)+w_{b}\hat{B}\left(v\right)+w_{ha}\hat{HA}\left(v\right)+w_{de}\hat{DE}\left(v\right).$$

where $S_{base}\left(v\right)$ denotes the basic score of KSGR; all quantities with tildes represent normalized structural features; and $w_{ks}$, $w_{r}$, $w_{g}$, $w_{b}$, $w_{ha}$, and $w_{de}$ are the weights of the corresponding structural features.

The contribution of each structural component in Equation (19) is further investigated through a component-wise ablation experiment in Section 5.7. In the complete implementation, KSGR selects branches according to the network Profile(G) = n,r,w,s, where n is the number of nodes, r denotes directed reciprocity, w is the proportion of the largest weakly connected component, and s is the proportion of the largest strongly connected component. Different networks are assigned to the sparse acyclic quota branch, the reciprocal-network tail-enhancement branch, the giant strongly connected component entropy-bridging branch, the structural diversity enhancement branch, or the core branch.

For any ranking score S, the rank normalization function is defined as follows:

$$\rho_{S}\left(v\right)=\frac{n-{\mathrm{rank}}_{S}\left(v\right)}{n-1}.$$

where $\rho_{S}\left(v\right)$ denotes the rank-normalized value of node v under the ranking score S; ${\mathrm{rank}}_{S}\left(v\right)$ is the ranking position of node v; and n is the number of nodes in the network.

Then, the KSGR score with the adaptive enhancement intensity parameter is defined as follows:

(20) $$KSGR_{\alpha }\left(v\right)=\alpha \rho_{S_{ad}}\left(v\right)+\left(1-\alpha \right)\rho_{S_{ref}^{\left(b\right)}}\left(v\right),0\leq \alpha \leq 1.$$

When α=1, the model recovers the formal KSGR ranking; when α=0, only the branch reference signal is retained. This parameter is used to examine the contribution of adaptive structural enhancement to spreading capability.

### 3.6. Theoretical Motivation and Branch-Selection Rationale

Although exact influence maximization under stochastic spreading models is generally difficult, the structural components of KSGR are motivated by the expected one- and two-step expansion process of the SI model adopted in this paper. Under the reverse-edge diffusion convention, the first-order in-neighborhood of a seed gives the immediate infection opportunity, while the second-order reverse neighborhood represents the next wave of potential exposure. Therefore, reverse local reachability and reverse diffusion efficiency approximate the early marginal spreading gain of a node. Hierarchical asymmetry distinguishes nodes whose reverse spreading potential is stronger than their forward outward-expansion structure, which is important when the edge direction represents following, citation, trust, or dependency rather than the actual direction of influence.

The reverse structural gravity term can be interpreted as a local approximation to the expected amount of reachable infectious mass within a limited reverse frontier. By multiplying reverse reachability and reverse k-shell, the mass term favors nodes that both cover many potential receivers and lie in a core reverse-spreading backbone; by introducing hop decay and structural distance, the gravity term penalizes remote or weakly connected frontiers. The bridging score further encourages nodes that connect low-clustering regions, and the Novelty/Overlap reranking step reduces redundant reverse two-hop coverage among selected seeds. Consequently, the proposed score is not a formal closed-form solution to the SI influence maximization problem, but a direction-consistent structural surrogate for selecting seeds with high early-stage reachability and complementary final-stage coverage.

The adaptive branch-selection rules are designed as a lightweight network profiling mechanism rather than as learned decision boundaries. Network size is used to distinguish small networks from large sparse graphs, reciprocity reflects the degree to which reverse and forward neighborhoods overlap, the LWCC ratio measures whether most nodes lie in a weakly connected backbone, and the SCC ratio measures the strength of directed cyclic connectivity. These four descriptors are inexpensive to calculate and correspond directly to the structural cases considered by KSGR: sparse acyclic expansion, reciprocal communication, giant strongly connected components, and structurally diverse weakly connected graphs. Because the thresholds are heuristic, this paper treats them as default profiling rules and evaluates their practical contribution through the adaptive enhancement parameter α. Since the branch-selection thresholds are manually specified profiling rules rather than learned parameters, their robustness is further examined through a threshold perturbation analysis in Section 5.6. Specifically, the cutoff values of the network size, reciprocity, LWCC ratio, and SCC ratio are independently increased or decreased by 10% and 20%, and the resulting branch assignments and ranking performance are compared with the original setting. For an interpretable node-level demonstration of the KSGR scoring mechanism on a small directed simulation network, see Appendix A.

## 4. SI Transmission Models and Evaluation Metrics

This paper adopts the SI spreading model to evaluate the spreading capability of influential nodes identified by different algorithms. The SI-based spreading evaluation is widely adopted in influential node identification studies because the ultimate objective of identifying influential nodes is to discover nodes with stronger spreading capability in network dynamics. Previous studies have also used diffusion-based experiments to evaluate whether ranking methods can effectively identify nodes that generate larger influence ranges under spreading processes [9]. Therefore, in this work, the SI model is employed as an external validation mechanism to measure the relative diffusion ability of nodes selected by different algorithms, rather than to reproduce the exact propagation process of a specific application scenario.

The spreading direction is consistent with the directed spreading setting used in the NEM and ImPageRank algorithms [9,25]. Initially, the selected seed nodes are set to the infected state, while all other nodes are in the susceptible state. At each time step, infected nodes infect their in-neighbors with a certain probability; that is, spreading proceeds along the direction of predecessors.

It should be noted that the reverse-edge diffusion setting adopted in this paper represents a structural propagation assumption rather than a universal description of all real-world diffusion processes. Similar assumptions are commonly used in directed influence analysis when the observed edge direction reflects relational dependency, attention receiving, or accessibility rather than direct spreading direction. The purpose of this evaluation is therefore to compare the relative effectiveness of ranking algorithms under the same directed propagation mechanism, rather than to reproduce the exact domain-specific diffusion dynamics of each dataset.

Consistent with the spreading convention, if  A points to B, then B can directly influence A.

(21) $$\beta_{th}=\frac{1}{\left\langle k_{in}\right\rangle }.$$

where $\left\langle k_{in}\right\rangle$ denotes the average in-degree of the network. For each algorithm, the Top-10 nodes with the highest scores are selected as the initial seeds. Multiple SI simulations are then conducted, and the average infection curve is obtained. In this paper, the area under the spreading curve AUC and the final infection scale are used to measure spreading capability.

The Top-10 setting used here is an experimental evaluation protocol rather than a restriction of KSGR. KSGR outputs a complete ranking list for all nodes, so the same ranking result can be directly used to select the Top-k nodes for any required seed-set size, such as 5, 10, or 50. This paper adopts Top-10 seeds because previous studies on influential node identification commonly evaluate the most highly ranked nodes by comparing Top-10 lists or the spreading ability of Top-10 seed sets under SI/SIR simulations [32,33,34]. Therefore, the Top-10 setting provides a concise and comparable benchmark for examining whether an algorithm can accurately identify the most influential nodes. To further address the influence of seed-set size, this paper also reports supplementary SI experiments on DD68 and tech-pgp using Top-5 and Top-20 seed nodes, while keeping the spreading direction, infection probability, maximum time steps, and number of independent simulations consistent with the main SI evaluation.

In addition to spreading capability, this paper introduces the largest weakly connected component (LWCC) attack experiment to evaluate the structural impact after node removal. Specifically, according to the node importance ranking produced by each algorithm, a certain proportion of high-scoring nodes is sequentially removed, and the size of the largest weakly connected component in the remaining network is calculated at each removal ratio. A faster decline in LWCC indicates that the removed nodes are more critical to the overall connectivity backbone of the network. This paper further calculates the normalized area under the LWCC curve, denoted as AUC_LWCC, as a comprehensive indicator of structural disruption. A smaller AUC_LWCC indicates stronger structural dismantling capability of the node identification method.

To compare the consistency and differences among the ranking results of different algorithms, this paper adopts Kendall’s τ correlation coefficient for ranking correlation analysis. Kendall’s τ characterizes the rank correlation between two ranking sequences by counting the numbers of concordant and discordant node pairs. A value of τ closer to 1 indicates that the rankings produced by the two algorithms are more consistent; a value closer to −1 indicates that the rankings are more opposite; and a value close to 0 indicates weak ranking correlation.

(22) $$\tau =\frac{N_{c}-N_{d}}{\sqrt{\left(N_{c}+N_{d}+T_{x}\right)\left(N_{c}+N_{d}+T_{y}\right)}}.$$

where $N_{c}$ and $N_{d}$ denote the numbers of concordant and discordant node pairs, respectively, and $T_{x}$ and $T_{y}$ are the tie corrections for the two ranking sequences.

## 5. Experimental Results and Analysis

### 5.1. Datasets

The six datasets cover sparse acyclic networks, large low-reciprocity networks, small high-reciprocity networks, and networks with structural diversity. Based on the network size, reciprocity, proportion of the largest weakly connected component, and proportion of the largest strongly connected component, KSGR assigns different networks to different structural branches: DD68 and DD687 enter the sparse acyclic quota branch; p2p-Gnutella04 enters the giant SCC entropy-bridging branch; email-Dept1 enters the reciprocal-network betweenness tail-enhancement branch; and physician_trust and tech-pgp enter the structural diversity enhancement branch. The statistical characteristics of the six datasets are summarized in Table 2.

### 5.2. SI Propagation Capability Experiment

**Figure 2:**
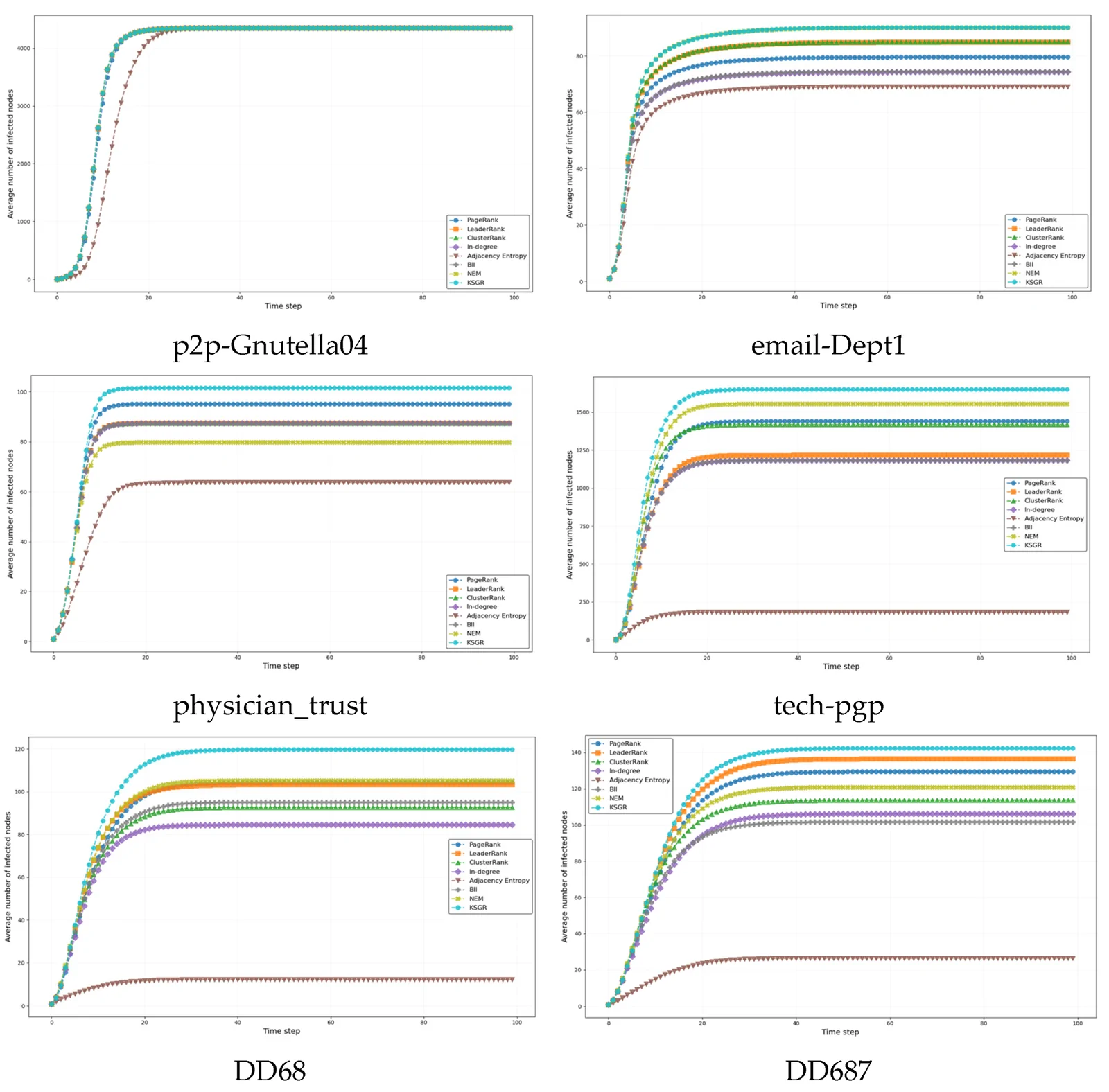
Average SI spreading curves of the Top-10 nodes identified by eight algorithms on six real-world directed networks. The six subfigures correspond to the p2p-Gnutella04, email-Dept1, physician_trust, tech-pgp, DD68, and DD687 datasets, respectively. The horizontal axis represents the spreading time step, and the vertical axis represents the average infection scale or infection ratio. Different curves correspond to PageRank, LeaderRank, ClusterRank, In-degree, Adjacency Entropy, BII, NEM, and KSGR, respectively. In the experiment, the 10 nodes with the highest scores produced by each algorithm are selected as the initial infected seeds. Spreading proceeds along the in-neighbor direction, and the final curve is obtained by averaging the results of multiple independent SI simulations.

As shown in Figure 2, in the p2p-Gnutella04 network, the spreading curves of all algorithms generally rise rapidly and then tend to stabilize, indicating that once influential nodes are activated in this network, the infection scale can expand rapidly within a short period. The KSGR curve remains at a relatively high level in both the early and middle stages of spreading. Compared with methods such as LeaderRank, ClusterRank, In-degree, BII, and NEM, KSGR exhibits a faster infection growth rate, and its final infection scale also remains at a superior level. The curve of Adjacency Entropy clearly lags behind, suggesting that relying solely on Adjacency Entropy is insufficient to capture effective spreading sources in this large-scale directed network. Overall, KSGR can cover large-scale spreading regions earlier, indicating that its reverse diffusion efficiency and structural gravity mechanisms are helpful for identifying core spreading nodes in p2p networks. In the email-Dept1 network, the KSGR curve consistently stays above those of the other algorithms throughout the spreading process, and its final infection scale is significantly higher than those of PageRank, In-degree, BII, and Adjacency Entropy. By combining the reverse local reachability, bridging capability, and structural diversity enhancement mechanism, KSGR can more effectively select Top-10 seed nodes with complementary coverage ranges. Therefore, it exhibits stronger spreading advantages in small-scale and highly reciprocal email communication networks. In the physician_trust network, the KSGR curve rises rapidly in the early stage of spreading and reaches the highest stable infection scale in the middle and later stages, clearly outperforming the other comparison algorithms. PageRank achieves the second-best performance, indicating that global link importance still plays a certain role in this network. In contrast, methods such as NEM, In-degree, and ClusterRank produce relatively lower final infection scales, suggesting that relying only on neighborhood entropy, in-degree, or local clustering information is insufficient to comprehensively characterize key spreading positions in the physician relationship network. The Adjacency Entropy curve remains at a clearly lower level, indicating its weak adaptability to the spreading direction and structural backbone of this type of network. The advantage of KSGR demonstrates that reverse k-shell, structural gravity, and bridging features can better identify cross-group spreading nodes. In the tech-pgp network, the differences among the spreading curves of different algorithms are relatively evident. The KSGR curve is located at the highest level, with a rapid spreading growth rate and the largest final infection scale. NEM ranks second, indicating that multi-order neighborhood information plays an important role in this network. PageRank and ClusterRank achieve medium-level performance, whereas LeaderRank, In-degree, and BII show relatively smaller spreading scales, and the Adjacency Entropy curve is far lower than those of the other methods. Since the tech-pgp network is large-scale and structurally sparse, ordinary local indicators are easily limited by insufficient coverage. In contrast, through reverse structural gravity and the structural diversity enhancement mechanism, KSGR can preferentially select nodes located in the spreading backbone and cross-regional connection positions, thereby showing clear advantages in this type of sparse large-scale network. In the DD68 network, the KSGR curve is generally at the highest level, and its final infection scale is significantly higher than those of most baseline algorithms. Methods such as LeaderRank, ClusterRank, PageRank, and NEM can also reach relatively stable spreading scales in the middle and later stages, but their overall curves remain lower than that of KSGR. In-degree and BII show relatively moderate performance, indicating that relying solely on the in-degree or iteratively accumulated influence is insufficient to distinguish key spreading sources in this sparse directed network. The Adjacency Entropy curve is the lowest, suggesting its insufficient adaptability to spreading along the in-neighbor direction. The performance of KSGR on this dataset indicates that, in sparse acyclic or weakly cyclic directed networks, reverse diffusion efficiency and reverse k-shell can effectively improve the spreading effectiveness of influential node ranking. In the DD687 network, the KSGR curve remains consistently ahead during the spreading process and achieves the largest final infection scale. LeaderRank and PageRank also show relatively good spreading performance, but there is still a certain gap compared with KSGR. ClusterRank, NEM, and In-degree are at an intermediate level, while BII and Adjacency Entropy show weaker spreading effects. This result indicates that, in sparse directed networks such as DD687, whether a node can expand to a broader second-order neighborhood along the reverse spreading direction is an important factor affecting the final spreading scale. By integrating reverse local reachability, reverse diffusion efficiency, and the structural diversity enhancement mechanism, KSGR can reduce spreading overlap among the Top-10 seed nodes, thereby achieving higher overall spreading gains.

As shown in Figure 2 and Table 3, KSGR achieves an average normalized SI AUC of 0.9747 and an average normalized final infection scale of 0.9871 across the six datasets, both of which are the highest among the eight algorithms. In datasets such as DD68, DD687, physician_trust, and tech-pgp, the spreading curves of KSGR are generally at a superior level. In p2p-Gnutella04 and email-Dept1, the spreading curves of KSGR are very close to those of the best baseline algorithms, while it maintains the best or tied-best performance in terms of the final infection scale. These results indicate that reverse diffusion efficiency, reverse structural gravity, and structural diversity enhancement can effectively improve the coverage capability of spreading seeds.

In contrast, Adjacency Entropy exhibits relatively weak overall spreading capability under the spreading setting adopted in this paper, suggesting that relying solely on Adjacency Entropy is insufficient to characterize diffusion potential along the in-neighbor direction. NEM performs strongly on most networks, but KSGR further improves the comprehensive spreading performance of the Top-10 seed set through bridging capability and the coverage redundancy reduction mechanism. In Figure 2, node markers and dashed line styles are used to distinguish different algorithms, highlighting the upper-bound spreading curves achieved by KSGR on most datasets.

#### Supplementary SI Propagation Experiment with Top-5 and Top-20 Seed Nodes

**Figure 3:**
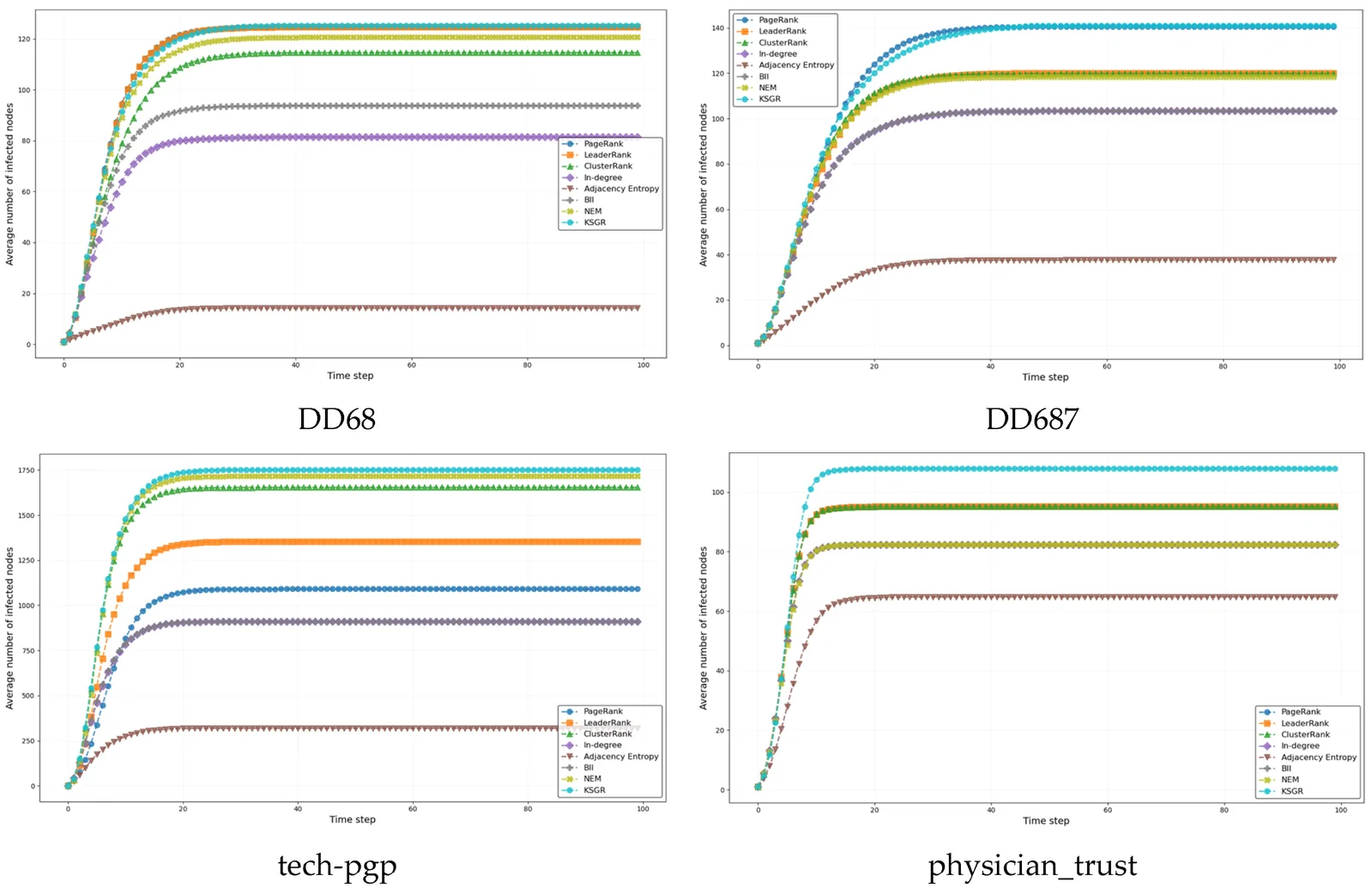
Average SI spreading curves on four networks under the Top-5 initial seed setting. The horizontal axis represents the spreading time step, and the vertical axis represents the average infected node ratio. The different curves correspond to PageRank, LeaderRank, ClusterRank, In-degree, Adjacency Entropy, BII, NEM, and KSGR.

**Figure 4:**
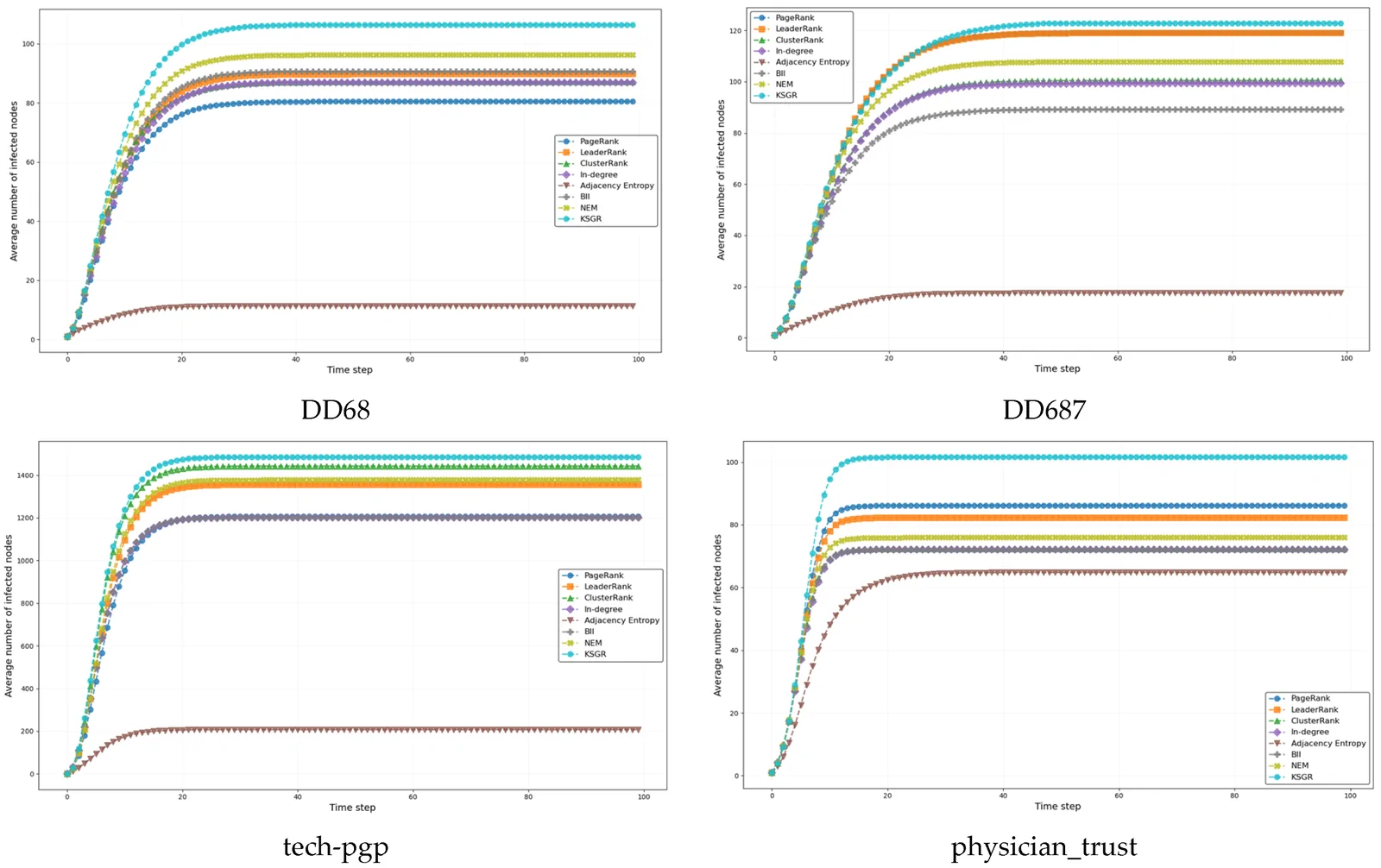
Average SI spreading curves on four networks under the Top-20 initial seed setting. The experimental protocol is the same as that used in Figure 3, except for the seed-set size.

To further examine whether the observed performance depends on the fixed Top-10 seed setting, four supplementary SI spreading experiments are conducted using different numbers of initially infected nodes to demonstrate the general applicability of KSGR. Specifically, the Top-5 and Top-20 seed settings are considered. DD68 is selected as a representative small-scale sparse directed network, while tech-pgp is selected as a representative large-scale sparse directed network; physician_trust and DD687 are also included as supplementary networks. All other experimental settings remain consistent with the main SI evaluation: spreading proceeds along the in-neighbor direction, the infection probability is set to the reciprocal of the average in-degree, and the maximum spreading time is 100 steps. For each ranking method, every node among the Top-5 or Top-20 ranked nodes is individually used as the sole initial infected source and independently simulated 50 times. The average spreading curve is first obtained for each individual source node, and these source-level curves are then averaged to produce the corresponding Top-k mean spreading curve for each method. The resulting Top-5 and Top-20 mean spreading curves are shown in Figure 3 and Figure 4, respectively.

The supplementary experimental results demonstrate that KSGR is not restricted to the Top-10 experimental setting and can directly generate seed sets of different sizes from the same complete node-ranking list. On the DD68, DD687, tech-pgp, and physician_trust networks, KSGR achieves the largest final spreading scale among all compared algorithms under both the Top-5 and Top-20 settings. Therefore, the Top-10 setting adopted in the main experiments should be regarded as a benchmark protocol for convenient comparison rather than a limitation of the proposed algorithm. These results further suggest that KSGR also performs well under other seed-set sizes, demonstrating its broad applicability and generalizability.

### 5.3. Kendall Correlation Analysis

To further compare the rank consistency among different node ranking methods, this paper employs Kendall’s τ to conduct correlation analysis on the node importance rankings generated by the eight algorithms. For each dataset, node importance rankings are first generated using PageRank, LeaderRank, ClusterRank, In-degree, Adjacency Entropy, BII, NEM, and KSGR. Then, within the same candidate node range, the Kendall correlation coefficient is calculated for the ranking results of every pair of algorithms, and an 8 × 8 correlation matrix is constructed.

**Figure 5:**
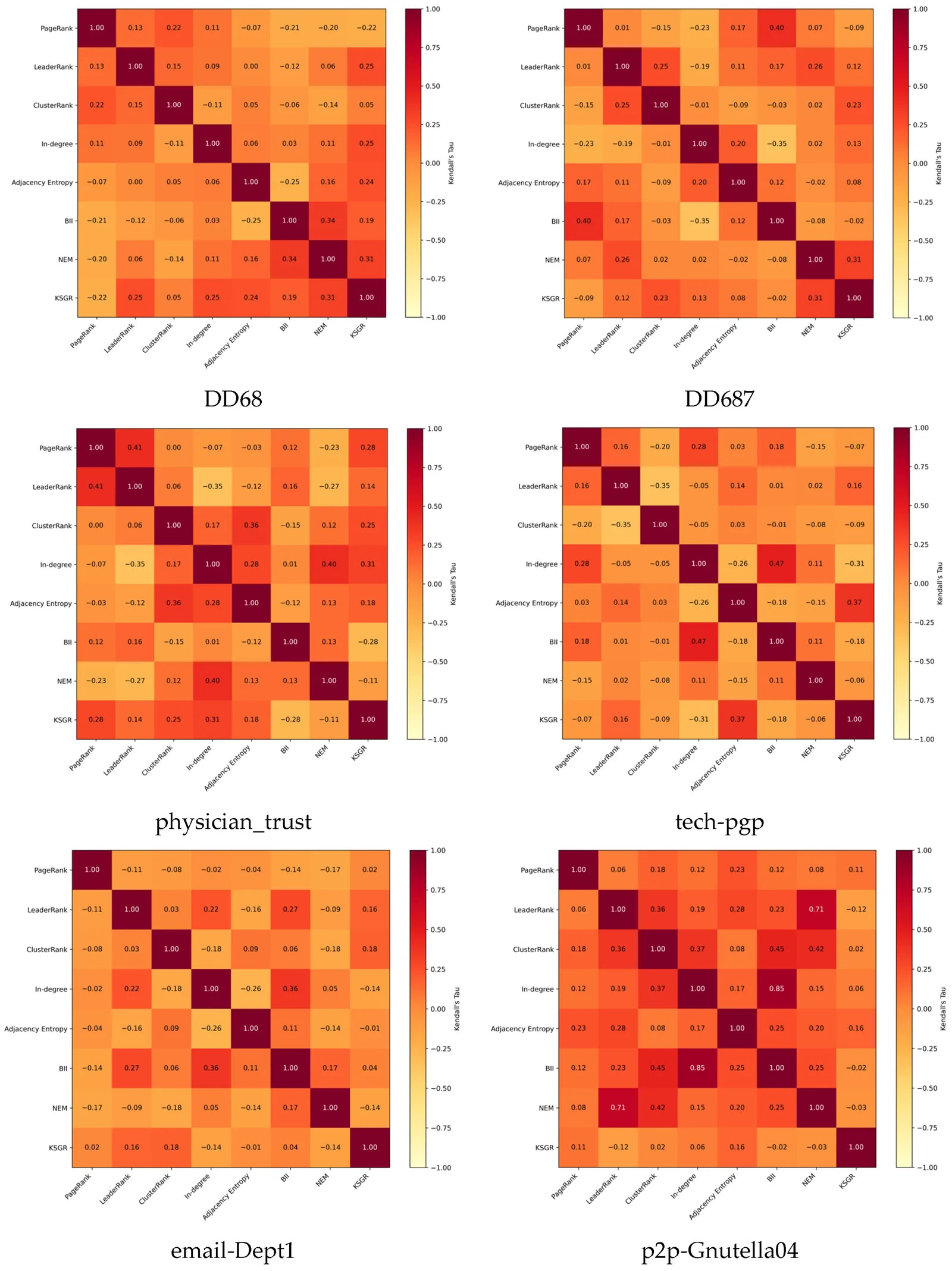
Kendall correlation heatmaps of the node ranking results generated by the eight algorithms on six real-world directed networks. The six subfigures correspond to the DD68, DD687, email-Dept1, p2p-Gnutella04, physician_trust, and tech-pgp datasets, respectively. Both the rows and columns of each matrix represent the eight ranking methods, namely PageRank, LeaderRank, ClusterRank, In-degree, Adjacency Entropy, BII, NEM, and KSGR. The color indicates the Kendall’s τ correlation coefficient between the ranking results of two algorithms. A color closer to the positive-correlation end indicates stronger ranking consistency between the two algorithms, whereas a color closer to the negative-correlation end or the neutral region indicates more evident differences in the structural preferences of the two methods for identifying influential nodes.

As shown in Figure 5, the Kendall correlation coefficients between KSGR and most baseline algorithms generally fall within a low-to-moderate range, and the correlation patterns vary markedly across datasets. This indicates that the node rankings produced by KSGR do not maintain a fixed level of consistency with any single traditional centrality method. In the sparse near-acyclic network DD68, KSGR exhibits weak positive correlations with NEM, LeaderRank, and In-degree, with a relatively higher correlation with NEM, suggesting that these methods share some degree of agreement in ranking nodes according to certain structural characteristics. Nevertheless, the overall correlations remain limited. In DD687, KSGR maintains positive correlations with NEM and ClusterRank, while showing weak negative correlations close to zero with PageRank and BII. This suggests that KSGR does not simply reproduce the ranking results of random-walk-based or iterative influence methods.

In p2p-Gnutella04 and email-Dept1, the Kendall coefficients between KSGR and the baseline algorithms are mostly close to zero, whereas tech-pgp and physician_trust exhibit mixed patterns involving both positive and negative correlations. These results indicate clear differences among the highly ranked nodes identified by different algorithms. Considering the structural mechanisms underlying these methods, such differences may be attributed to their distinct emphases on reverse spreading-source positions, local coverage ranges, core hierarchy, and bridging structures. KSGR jointly incorporates reverse diffusion efficiency, reverse k-shell, structural gravity, bridging capability, and the Novelty/Overlap reranking mechanism used in the corresponding enhancement branches. Consequently, it not only shares certain structural ranking information with some traditional methods, but also introduces ranking signals that differ from those of conventional centrality and random-walk-based approaches. It should be noted that Kendall correlation primarily reflects ranking consistency and differentiation and, by itself, cannot establish the superiority of ranking performance. The spreading effectiveness of the KSGR ranking should therefore be evaluated in conjunction with the SI spreading experiments and the LWCC node-removal experiments.

### 5.4. LWCC Network Robustness Experiment

It should be noted that the LWCC metric adopted in this work originates from network-dismantling research for undirected networks, and it is introduced here primarily to offer a computationally efficient reference for evaluating the backbone-destruction capability of detected influential nodes. Since LWCC disregards edge directional information when computing connected components, it cannot fully capture the directed topological characteristics of real-world directed networks. Consequently, LWCC-based results are only suitable as one reference perspective for structural-impact assessment, rather than a complete evaluation of directed-network resilience. In future follow-up investigations, robustness metrics built upon the largest strongly-connected component (SCC) and directed reachability will be further incorporated, so as to achieve a more comprehensive assessment of the network-disruption performance for directed networks.

**Figure 6:**
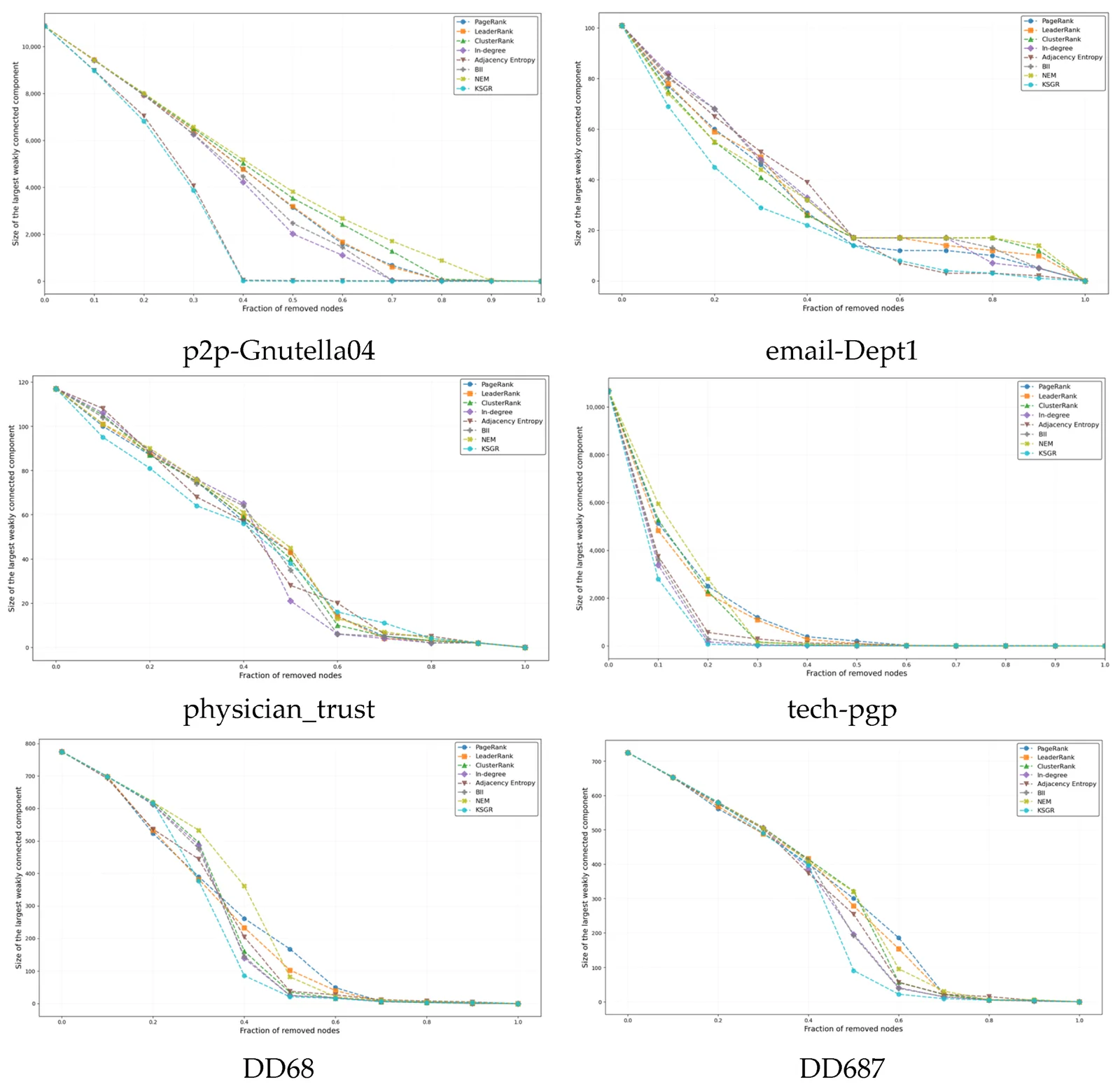
Changes in the largest weakly connected component (LWCC) after removing high-ranking nodes identified by different algorithms on six real-world directed networks. The six subfigures correspond to the p2p-Gnutella04, email-Dept1, physician_trust, tech-pgp, DD68, and DD687 datasets, respectively. The horizontal axis represents the proportion of nodes progressively removed according to node importance ranking, and the vertical axis represents the normalized size of the largest weakly connected component. A faster decline in the curve or a lower overall curve position indicates that the high-ranking nodes preferentially identified by the algorithm play a stronger supporting role in the weakly connected backbone of the network and that removing these nodes causes more severe structural disruption to the network.

To evaluate the impact of the nodes identified by each algorithm on the network connectivity backbone, this paper conducts the LWCC node-removal experiment. Unlike the single-point structural impact evaluation that removes only the Top-10 nodes, the LWCC experiment sequentially removes high-ranking nodes according to the ranking produced by each algorithm and calculates the size or proportion of the largest weakly connected component in the remaining network under different removal ratios. Figure 6 shows the changing trend of LWCC during the removal process. Table 4, Table 5, Table 6, Table 7, Table 8 and Table 9 further report the normalized LWCC values after removing 10%, 30%, and 50% of nodes, and calculate AUC_LWCC over the interval from 0% to 50% as a quantitative comparison metric. A faster decline in the curve or a smaller AUC_LWCC indicates that the nodes preferentially identified by the algorithm are more capable of disrupting the weakly connected structure of the network.

As shown in Figure 6, in the p2p-Gnutella04 network, the LWCC size decreases as the proportion of removed high-ranking nodes increases for all algorithms, but the rate of decline differs significantly. The KSGR curve declines relatively rapidly overall. In particular, after approximately 30–40% of nodes are removed, the largest weakly connected component quickly approaches a disintegrated state, indicating that the nodes preferentially identified by KSGR play a strong supporting role in the weakly connected backbone of the network. In contrast, the LWCC curves of PageRank, LeaderRank, ClusterRank, In-degree, BII, and NEM decline more slowly, suggesting that the high-scoring nodes identified by these methods have weaker destructive effects on the overall weakly connected structure. This result indicates that KSGR is not only suitable for spreading maximization tasks, but can also effectively locate key structural nodes in large-scale sparse networks. In the email-Dept1 network, the KSGR curve declines the fastest in the early stage of node removal. After removing 10% and 30% of the high-ranking nodes, the remaining LWCC size is significantly lower than that of most baseline algorithms, indicating that KSGR can identify key nodes that maintain the weakly connected backbone of the email network at an early stage. As the removal ratio continues to increase, the curves of different algorithms gradually become closer. This suggests that, due to the small scale and high reciprocity of this network, when a large number of nodes are removed, the overall network structure tends to become fragmented, thereby reducing the differences among algorithms. Overall, the rapid dismantling capability exhibited by KSGR under low removal ratios is of greater practical significance, demonstrating that its bridging capability and structural diversity enhancement term can effectively identify critical connecting nodes in communication networks.

In the physician_trust network, the KSGR curve remains at a relatively low level throughout the node-removal process, indicating that the nodes identified by KSGR have a strong impact on the weakly connected structure of the network. It should be noted that, at certain removal ratios, methods such as In-degree may exhibit a locally faster decline. However, from the perspective of the overall curve area, KSGR still achieves better comprehensive structural disruption performance. This suggests that in small-scale and relatively dense relational networks, a single indicator may perform well at a certain removal stage, but its stability is insufficient. By simultaneously considering the reverse reachability, shell position, and bridging structure, KSGR can more stably locate the nodes that support the network connectivity backbone and therefore achieves better overall robustness evaluation. In the tech-pgp network, the LWCC curve declines very rapidly overall, indicating that although this network is relatively large, its weakly connected backbone is highly sensitive to a small number of key nodes. KSGR causes a substantial decrease in LWCC size at the early stage of node removal, and after approximately 30% of nodes are removed, the largest weakly connected component of the network is nearly completely dismantled, demonstrating strong structural dismantling capability. In contrast, methods such as PageRank, LeaderRank, and NEM retain larger LWCC sizes in the early stage, suggesting that the nodes identified using these methods are not necessarily located at the most critical connectivity-supporting positions. The advantage of KSGR in this network indicates that reverse structural gravity and bridging scores can effectively capture backbone nodes in large-scale sparse networks.

In the DD68 network, the LWCC curves of all algorithms generally exhibit a stepwise decreasing trend. Among them, KSGR declines more rapidly after a moderate removal ratio and achieves the best performance in terms of the overall curve area. This phenomenon indicates that there exist several critical connecting nodes in the DD68 network; once these nodes are preferentially removed, the weakly connected backbone quickly breaks apart. Although methods such as PageRank, LeaderRank, In-degree, and BII can also identify some important nodes, they still retain relatively large connected components when approximately 30% of nodes are removed, suggesting that their ranking results are less concentrated than KSGR in locating structurally supportive nodes. The advantage of KSGR reflects the effectiveness of reverse diffusion and shell-backbone information in structural disruption experiments on sparse directed networks. In the DD687 network, the LWCC decline of different algorithms is relatively similar in the early stage of node removal. However, as the removal ratio increases, the KSGR curve decreases more substantially. In particular, after approximately 50% of nodes are removed, the remaining largest weakly connected component is significantly smaller than those of most baseline algorithms. This result indicates that, in the DD687 network, key structural nodes are not determined solely by first-order degree or random-walk-based importance, but require comprehensive identification by combining the spreading direction, core backbone, and cross-regional connectivity. Through the adaptive structural enhancement mechanism, KSGR preferentially selects nodes that provide stronger support for the weakly connected backbone and therefore exhibits stronger network dismantling capability in the middle and later stages of node removal.

As shown in Figure 6 and Table 4, Table 5, Table 6, Table 7, Table 8 and Table 9, the normalized AUC_LWCC values of KSGR based on the six datasets are 0.2755, 0.3453, 0.2289, 0.2171, 0.3160, and 0.0698, respectively, all of which are relatively low or the lowest values among the corresponding datasets. When 10%, 30%, and 50% of high-ranking nodes are removed, the LWCC ratios obtained by KSGR are also generally lower than those of most baseline algorithms. This indicates that KSGR can not only improve the coverage effectiveness of spreading seeds, but also preferentially identify nodes that provide strong support for the weakly connected backbone.

From the perspective of individual datasets, the tabular results for DD68 and DD687 show that, after removing 50% of nodes, KSGR reduces the normalized LWCC to 0.0271 and 0.1255, respectively, both lower than those of the other baseline algorithms. This indicates that reverse diffusion and shell-backbone features in sparse acyclic structures are helpful for locating critical connecting nodes. In large-scale networks such as p2p-Gnutella04 and tech-pgp, the AUC_LWCC values of KSGR are 0.2289 and 0.0698, respectively, reflecting the adaptability of structural gravity and bridging scores to large-scale sparse networks. In email-Dept1 and physician_trust, KSGR still maintains a relatively low LWCC curve area; however, due to the small scale and high reciprocity of these networks, the results of different algorithms gradually converge under high removal ratios. Overall, the LWCC results show that KSGR possesses good structural robustness evaluation capability, in addition to spreading capability.

### 5.5. Parameter Sensitivity and Robustness Analysis

To examine the contribution of the adaptive structural enhancement module, this paper conducts a sensitivity analysis on the parameter α. Here, α = 0 indicates that only the reference signal of the corresponding branch is retained, whereas α = 1 indicates that the formal KSGR ranking is restored. In this paper, SI spreading curves under different values of α are plotted based on the six datasets, and the average normalized AUC and final infection scale are calculated.

**Figure 7:**
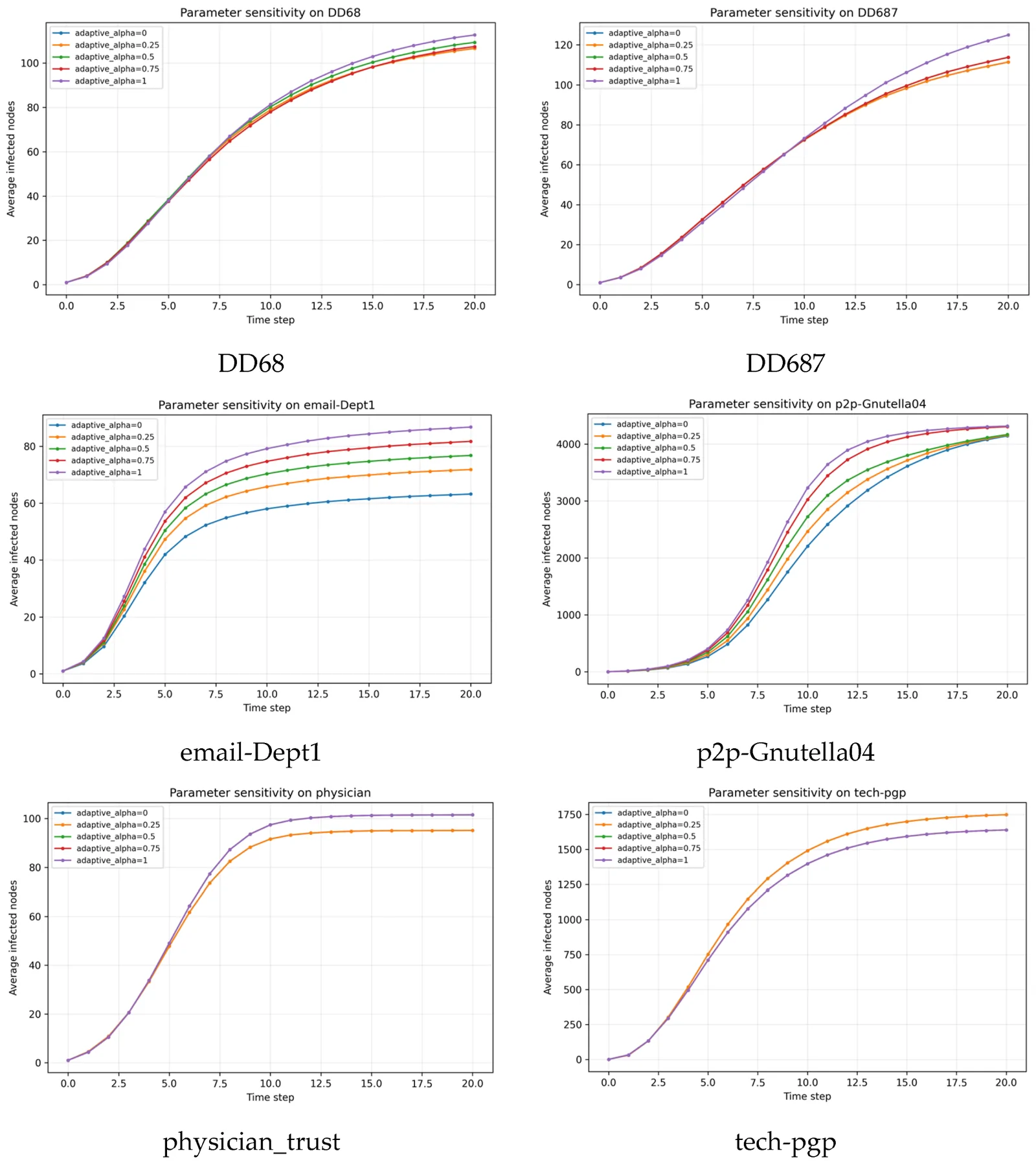
Effect of the adaptive enhancement parameter α on the SI spreading curves of KSGR on six real-world directed networks. The six subfigures correspond to the DD68, DD687, email-Dept1, p2p-Gnutella04, physician_trust, and tech-pgp datasets, respectively. The horizontal axis represents the spreading time step, and the vertical axis represents the average infection scale or infection ratio. Different curves correspond to different values of α, including α = 0, 0.25, 0.5, 0.75, and 1.00. Here, α = 0 indicates that only the reference ranking signal of the corresponding branch is retained, whereas α = 1 indicates that the complete KSGR ranking is restored. This figure is used to examine the contribution of the adaptive structural enhancement module to spreading capability and stability.

As shown in Figure 7, based on the DD68 dataset, the curves corresponding to different values of α show only minor differences in the early stage, indicating that the propagation initiation stage is mainly affected by the coverage range of the initial seeds. As spreading proceeds, the curve for α = 1 gradually achieves a higher infection scale, suggesting that the complete structural enhancement module can further improve subsequent diffusion capability in sparse directed networks. On the DD687 dataset, α = 1 performs best in the middle and later stages, and its final infection scale is higher than those obtained under other parameter settings. This indicates that, in this sparse network, the complete KSGR ranking can better exploit reverse diffusion, structural gravity, and coverage complementarity information, thereby improving the overall spreading effect of the seed set. Based on the email-Dept1 dataset, the curves generally shift upward as α increases. The spreading scale reaches the highest level when α = 1, whereas the curve for α = 0 is clearly lower. This result suggests that relying only on the branch reference signal is insufficient to characterize key spreading nodes in a highly reciprocal email network, while the complete structural enhancement module can significantly improve spreading coverage capability. Based on the p2p-Gnutella04 dataset, all curves rise rapidly and then tend to stabilize, while higher α values lead to faster growth in the middle stage of spreading. Among them, α = 1 maintains a relatively superior level. This indicates that the complete structural enhancement module helps select nodes located in the core backbone and efficient diffusion positions in large-scale directed networks. Based on the physician_trust dataset, the curve for α = 1 is generally higher than those for lower α values, and its final infection scale is larger. Since this network is small in scale and has strong local clustering, complete structural enhancement helps reduce coverage redundancy among seed nodes and improve the spreading complementarity of the Top-10 seed set. On the tech-pgp dataset, α = 0.25 is slightly higher than α = 1 in the later stage, indicating that weaker enhancement may sometimes preserve local spreading advantages in extremely sparse large-scale networks. However, from the overall trend, α = 1 still maintains strong spreading capability. Combined with the average results across the six datasets, the complete structural enhancement module demonstrates better overall stability and adaptability.

As shown in Figure 7 and Table 10, in DD68, DD687, email-Dept1, p2p-Gnutella04, and physician_trust, the spreading curves generally shift upward as α increases from 0 to 1, especially in the middle and later time steps. This indicates that the complete adaptive enhancement module can improve the spreading coverage capability of the candidate influential node set under different network structures. The curve performance based on tech-pgp shows a certain particularity: α = 0.25 achieves a higher infection scale than α = 1 in the later stage. This suggests that in large-scale networks that are extremely sparse and have a very low proportion of strongly connected components, the reference branch signal may still preserve some favorable local spreading advantages. However, according to the average results across the six datasets in Table 10, as α increases from 0 to 1, the average normalized AUC increases from 0.9013 to 0.9899, and the average normalized final infection scale increases from 0.9105 to 0.9896. Thus, α = 1 still achieves the best overall result. Therefore, the complete adaptive enhancement module is an important source of the stable spreading performance of KSGR.

**Figure 8:**
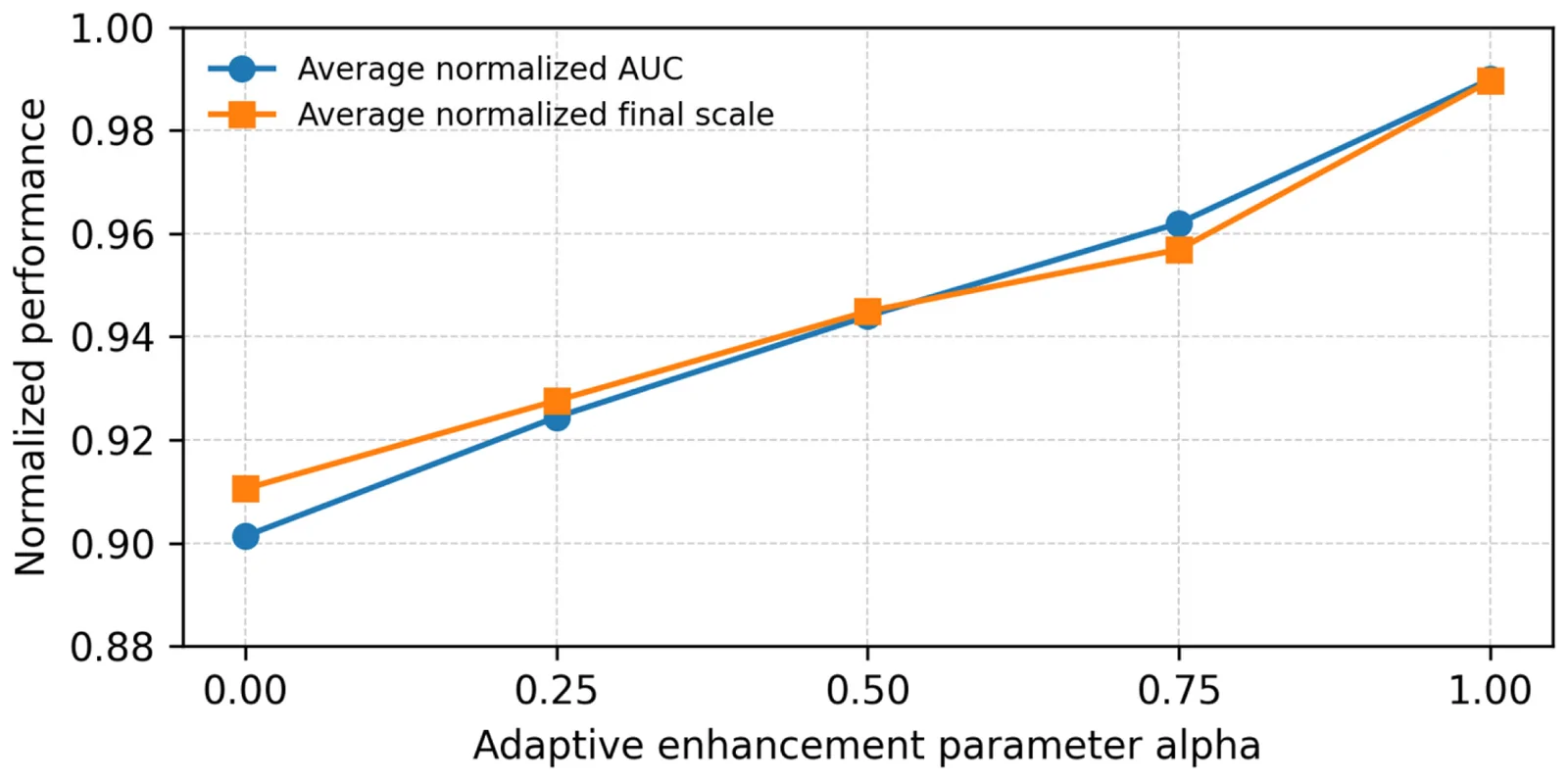
Average sensitivity trend of KSGR with respect to the adaptive enhancement parameter alpha. The two curves summarize the average normalized AUC and average normalized final infection scale over the six datasets reported in Table 10.

To provide a more quantitative sensitivity evaluation, the average results in Table 10 are further analyzed in terms of marginal gains and monotonicity. When alpha increases from 0 to 1, the average normalized AUC increases from 0.9013 to 0.9899, corresponding to an absolute improvement of 0.0886 and a relative improvement of approximately 9.83%. The average normalized final infection scale increases from 0.9105 to 0.9896, corresponding to an absolute improvement of 0.0791 and a relative improvement of approximately 8.69%. The performance indicators show a stable positive relationship with alpha. The Pearson correlation coefficients between alpha and the average normalized AUC and final infection scale are 0.9974 and 0.9855, respectively. Since both indicators increase strictly over the tested alpha values, the rank-order trend is monotonic. This suggests that KSGR is not unstable under small parameter changes; instead, stronger incorporation of the complete structural enhancement module generally leads to better spreading performance. The corresponding average sensitivity trends are illustrated in Figure 8.

Table 11 shows that the performance gain is positive in every adjacent alpha interval. The largest marginal increase appears in the interval from alpha = 0.75 to alpha = 1.00, indicating that the final integration of reverse reachability, reverse diffusion efficiency, reverse k-shell, structural gravity, bridging capability, and diversity-aware reranking is still useful after the branch reference signal has already been incorporated. Therefore, alpha = 1 is adopted as the default setting in the formal KSGR implementation.

Regarding the branch-selection thresholds, the network profile variables, including the network size, reciprocity, LWCC ratio, and SCC ratio, are used only to select the enhancement branch, whereas the final ranking is still determined by normalized structural features and the alpha-controlled blending mechanism. Therefore, the alpha sweep can also be interpreted as a practical robustness test of the dependence on branch-specific reference signals: alpha = 0 corresponds to using only the selected branch reference signal, while alpha = 1 corresponds to the complete KSGR score. The fact that alpha = 1 achieves the best average result indicates that the final ranking does not rely excessively on a single heuristic branch rule.

It should be noted that this sensitivity analysis does not exhaustively enumerate all possible threshold perturbations for branch selection. A full threshold-ablation experiment would require rerunning the complete ranking and SI simulation pipeline under multiple alternative network-profiling rules, such as ±10% and ±20% perturbations of the reciprocity, LWCC-ratio, and SCC-ratio thresholds. Nevertheless, the present alpha-based sensitivity analysis provides direct evidence that the adaptive enhancement intensity is stable across the six datasets and that the complete KSGR formulation is preferable to the branch-only version.

While α sensitivity evaluates the influence of enhancement intensity, the robustness of manually defined branch-selection thresholds is further investigated in Section 5.6.

### 5.6. Threshold Robustness Analysis of Adaptive Branch Selection

Since the adaptive branch-selection mechanism relies on manually defined network-profile thresholds, the original cutoff values used by KSGR are summarized in Table 12. These thresholds determine the selection of different structural branches according to network size, reciprocity, weakly connected component ratio, and strongly connected component ratio.

To evaluate the robustness of these heuristic thresholds, each threshold group was independently perturbed by ±10% and ±20% while keeping the other groups unchanged. Detailed perturbed cutoff values are provided in Appendix B. The corresponding perturbation settings are summarized in Table 13.

**Figure 9:**
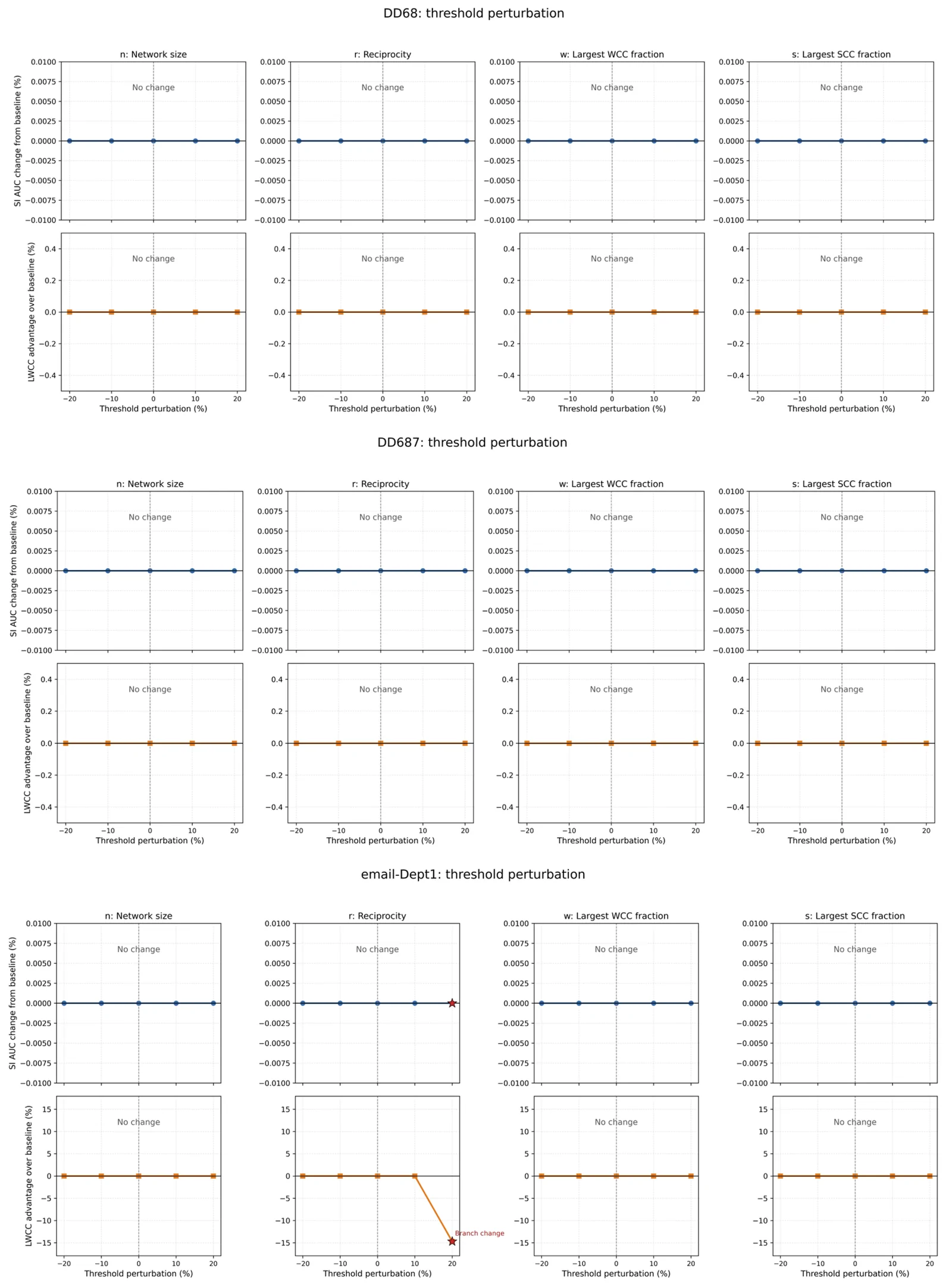

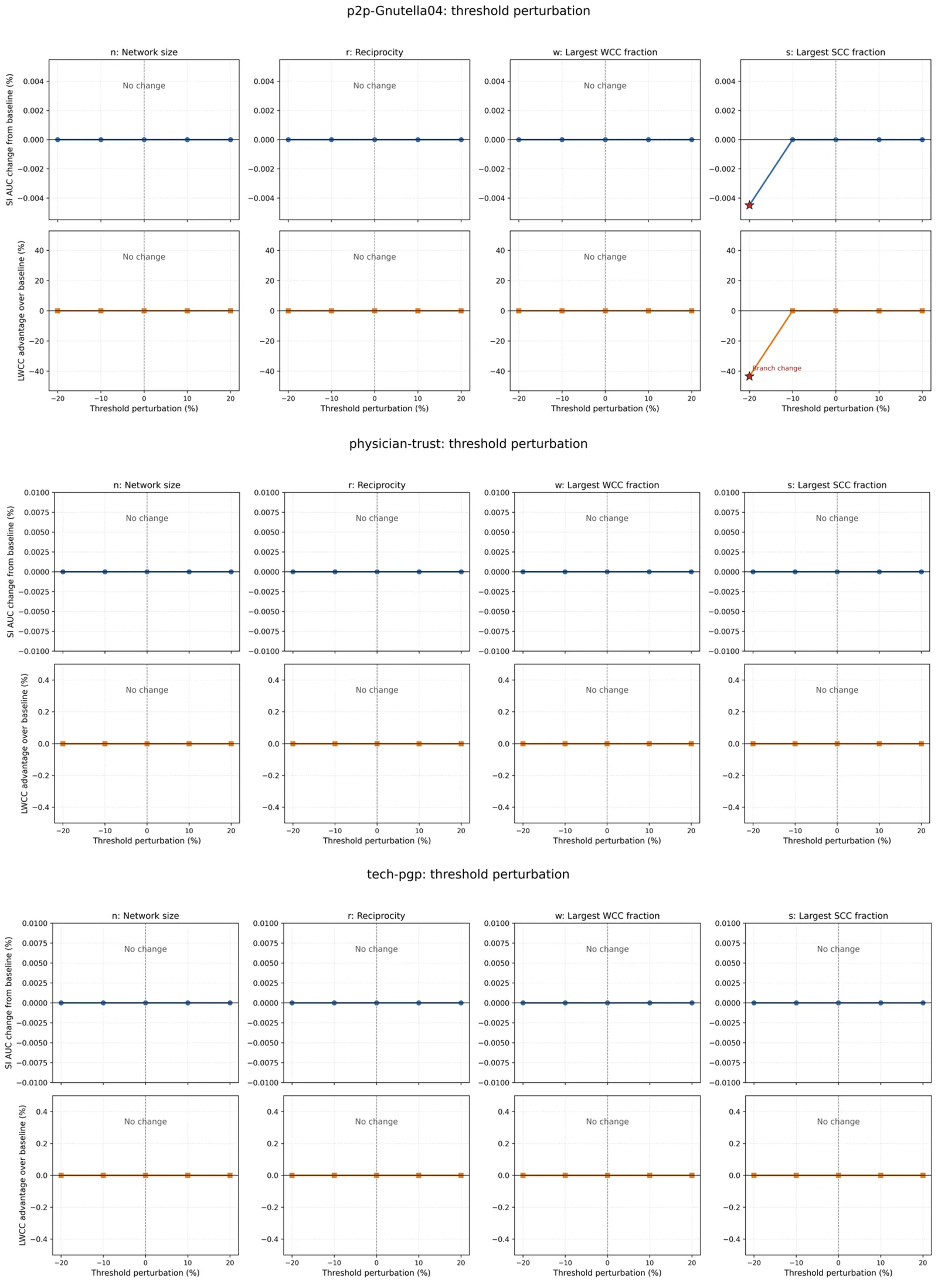
Branch stability, SI variation, and LWCC variation over the six datasets. Red stars indicate threshold perturbation settings that cause the adaptive branch assignment of KSGR to differ from that under the original threshold configuration; they do not indicate statistical significance, optimality, or outliers.

The resulting branch stability, SI variation, and LWCC variation across the six datasets are shown in Figure 9.

As shown in Figure 9, across all 102 perturbation settings, only two cases produce different branch assignments compared with the original configuration. This indicates that the adaptive branch-selection mechanism is generally stable under moderate threshold variations. The two branch-switch cases occur in p2p-Gnutella04 and email-Dept1, where the network profiles are close to the predefined boundaries.

Although branch switching may cause noticeable variation in the structural robustness evaluation, the SI-based spreading performance remains highly stable. This suggests that the threshold rules mainly influence structural enhancement selection rather than the basic spreading ability captured by the core ranking mechanism.

### 5.7. Component-Wise Ablation Study

To investigate the contribution of individual structural components in Equation (19), a leave-one-component-out ablation experiment is conducted. Each component is removed separately while the remaining weights are renormalized to maintain the original score scale.

Table 14 summarizes the correspondence between the structural components in Equation (19) and the corresponding ablation variants. Each ablation experiment removes one structural term from the complete KSGR model while keeping the remaining components unchanged. To ensure a fair comparison, the weights of the remaining terms are renormalized after removing a component so that the overall score scale remains consistent with the original formulation. All ablated variants use the same datasets, propagation settings, and Top-10 influential node selection strategy as the complete KSGR model. Therefore, the performance difference between each ablated variant and the full model reflects the individual contribution of the removed structural component. Specifically, the six ablation variants directly correspond to the six structural terms in Equation (19), including the reverse k-shell, reverse local reachability, reverse structural gravity, bridging capability, hierarchical asymmetry, and reverse diffusion efficiency.

In the ablation experiments, each structural component was removed individually from the complete KSGR model. Positive SI loss indicates that removing the component reduces spreading identification capability.

**Figure 10:**
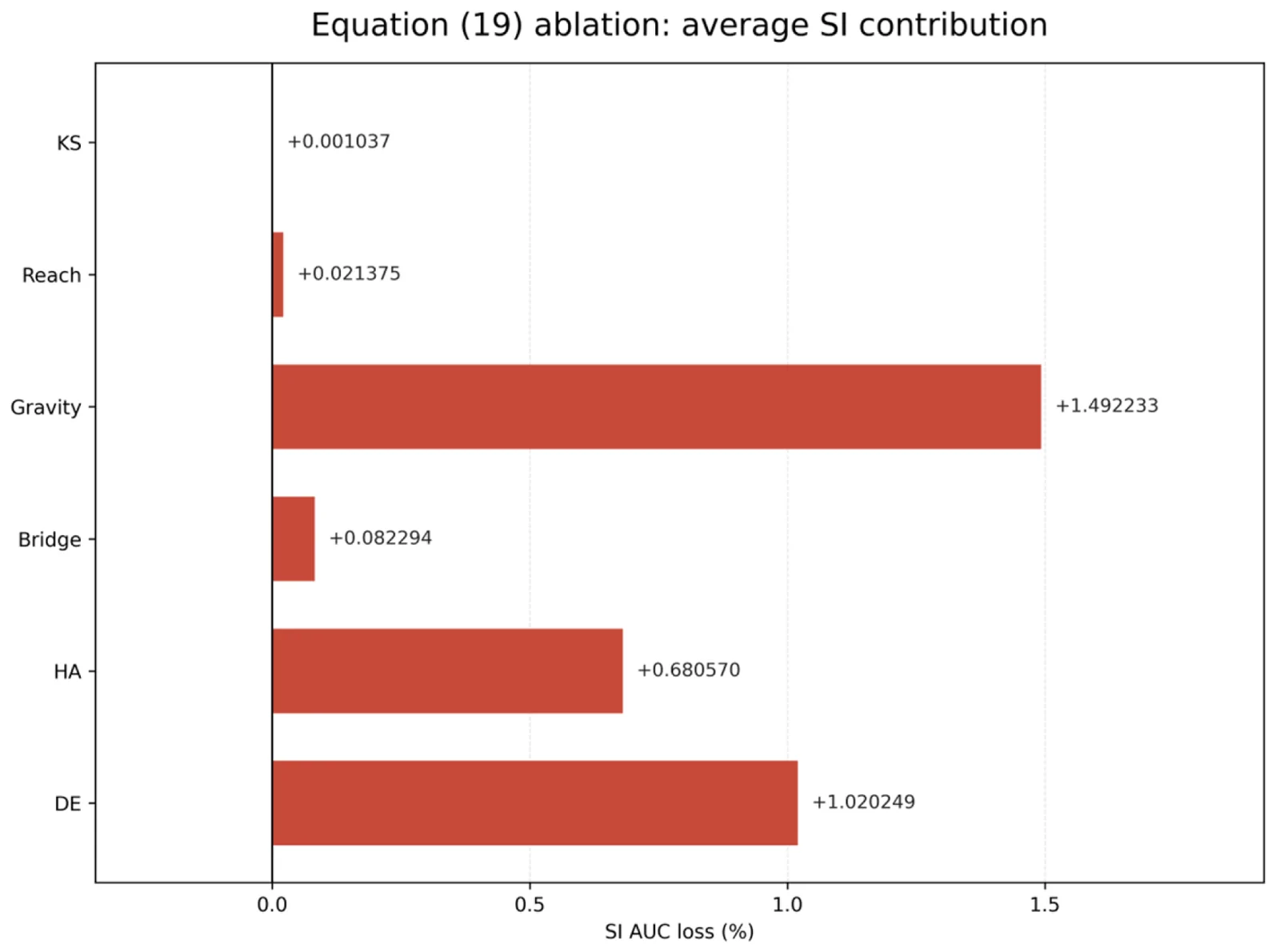
Component-wise ablation of the core score in Equation (19).

To investigate the contribution of each component in Equation (19), a component-wise ablation experiment is conducted by removing each structural term individually. Figure 10 reports the average SI AUC degradation across six networks. The results demonstrate that different components contribute unequally to the spreading influence identification capability of KSGR. Among all components, removing reverse structural gravity leads to the largest performance degradation, with an average SI AUC loss of 1.492%, indicating that global structural attraction information plays a crucial role in identifying influential nodes. Reverse diffusion efficiency also provides a significant contribution, as its removal results in a 1.020% decrease in the SI AUC. In addition, hierarchical asymmetry contributes to capturing directional structural differences, leading to a degradation of 0.681% after removal. In contrast, reverse k-shell and reverse reachability show relatively smaller individual effects, suggesting that their information may overlap with other structural features and mainly provides complementary structural cues. Although the removal of bridging capability causes limited SI degradation (0.082%), its primary contribution is reflected in network dismantling performance rather than spreading capability.

### 5.8. Complexity Analysis

The main computational cost of KSGR comes from reverse two-hop neighborhood statistics, reverse k-shell decomposition, reverse structural gravity, bridging score calculation, and structural diversity reranking. Let N and M denote the numbers of nodes and directed edges, respectively. The reverse in-neighborhood lists can be constructed in O(N + M) time and O(N + M) memory. Reverse k-shell decomposition on the reversed graph also requires O(N + M) time. Reverse two-hop statistics and the H = 2 structural gravity calculation are bounded by the total number of visited reverse frontier relationships; in sparse graphs, this cost is close to linear in M, while in the worst case, it can be written as O(Σu din(u)dout(u)) because the in-neighborhood of a node may be repeatedly visited through its outgoing incidences.

The bridging score is computed on the undirected projection. With adjacency-set operations, its local clustering component costs O(Σv du(v)^2) in a straightforward implementation, where du(v) is the undirected degree of node v; this term is typically moderate for the sparse networks considered in this paper and can be accelerated by standard triangle-counting or sparse-matrix operations. For structural diversity reranking, if L candidate nodes are retained in the preliminary pool and k seeds are selected, the additional coverage and overlap terms require O(k L C_avg) set operations, where C_avg is the average reverse two-hop coverage footprint size in the candidate pool. Since k = 10 in the experiments and L is much smaller than N, this reranking step does not dominate the total cost.

Overall, KSGR is more expensive than pure first-order measures such as In-degree, but avoids all-pairs shortest paths and does not require iterative convergence over the whole graph. Its memory consumption is O(N + M) if coverage footprints are generated on demand or O(N + M + Σv|Cv|) if all reverse two-hop coverage sets Cv are cached for faster reranking. Therefore, the scalability based on million-node networks should be further validated using optimized sparse data structures, parallel reverse-neighborhood traversal, and runtime/memory profiling; such large-scale engineering evaluation is beyond the current experimental scope.

## 6. Discussion

Although KSGR adopts a unified reverse-edge diffusion convention, the semantic meaning of directed edges varies across applications. Future studies may incorporate domain-specific propagation mechanisms, weighted edges, temporal information, or adaptive diffusion models to further improve the applicability of influential node identification in heterogeneous directed networks.

## 7. Conclusions

This paper proposes KSGR, an influential node identification algorithm for directed networks. The algorithm is designed for directed network scenarios in which information spreads along the in-neighbor direction and integrates the reverse diffusion efficiency, hierarchical asymmetry, directed reverse k-shell, reverse structural gravity, bridging score, and a structural diversity enhancement mechanism to characterize nodes’ local diffusion potential, structural backbone position, and coverage complementarity. The adopted reverse-edge propagation convention represents a specific directed spreading assumption, and its applicability may depend on the semantic meaning of the edge direction in different types of real-world networks.

Experiments on six real-world directed networks show that, under the propagation convention and SI simulation settings adopted in this study, KSGR achieves the highest average normalized AUC and final infection scale in the Top-10 seed experiments among the selected comparison methods, indicating its effectiveness in identifying influential nodes with strong spreading potential under the considered experimental setting. Kendall correlation experiments further show that KSGR does not simply overlap with traditional centrality, random-walk-based, and neighborhood-entropy-based methods, but produces differentiated ranking results by integrating multiple structural signals. The LWCC node-removal experiments show that KSGR achieves generally favorable normalized AUC_LWCC values across the six datasets, providing evidence that the highly ranked nodes have considerable influence on the weakly connected backbone. However, because LWCC does not preserve the edge direction, these results should be interpreted as an evaluation of weak-connectivity disruption rather than a complete characterization of directed-network robustness.

The choice of ten initial seeds should therefore be interpreted as a commonly used benchmark setting rather than as a fixed output size of the proposed method. In practical applications, users can obtain the required number of influential nodes by selecting the first k nodes from the complete KSGR ranking list.

Parameter sensitivity experiments show that the complete adaptive enhancement formulation achieves the best average spreading performance when alpha = 1 among the tested settings, and the average normalized AUC and final infection scale increase monotonically over the examined alpha values. These results support the practical effectiveness of the complete KSGR framework. Cross-network validation would provide additional evidence regarding the generalizability of the observed performance and robustness.

Moreover, threshold perturbation experiments demonstrate that the adaptive branch-selection mechanism is robust to moderate variations of profiling thresholds, while component-wise ablation verifies the complementary roles of reverse structural gravity, bridging capability, and diffusion-related features.

## 8. Research Prospects

From the perspective of improving the KSGR algorithm itself, future work may focus on enhancing the model’s adaptability and scalability. The current KSGR still adopts fixed feature weights and rule-based branch selection. In the future, automatic parameter learning, Bayesian optimization, or learning-to-rank mechanisms can be introduced to dynamically adjust the weights of different structural features according to the network size, reciprocity, strongly connected structure, and spreading feedback. Meanwhile, the algorithm can be extended to dynamic, weighted, and multilayer directed networks and combined with incremental updating, parallel computing, and approximate neighborhood sampling to improve its computational efficiency and application stability in large-scale networks.

From the perspective of influential node identification in directed networks, future research should further strengthen the consistency between spreading semantics and evaluation objectives. Edges in different directed networks may represent following, citation, communication, trust, or dependency relationships, and their real influence directions are not completely identical. Therefore, it is necessary to characterize in-neighborhood spreading, out-neighborhood diffusion, bidirectional feedback, and higher-order group relationships in combination with specific scenarios. In addition, influential node identification can be extended from single-objective spreading maximization to a multi-objective problem that simultaneously considers spreading coverage, structural robustness, ranking monotonicity, and seed set complementarity, thereby improving the applicability of the method in scenarios such as public opinion control, risk protection, and recommender systems.

Several limitations should also be acknowledged. First, the present experiments use six real-world directed networks, which provide initial evidence across sparse, reciprocal, and structurally diverse cases, but they do not fully cover very large citation networks, web graphs, social-media graphs, or temporal directed networks. Second, the comparison includes representative classical and recent centrality methods, but does not yet include all modern graph-neural-network-based ranking models, collective-influence variants, or the latest hybrid community-aware methods such as [20,29]. Third, the branch thresholds in KSGR are currently rule-based and the complexity analysis is mainly asymptotic; future work should add cross-dataset parameter transfer tests, runtime comparisons, and memory measurements on million-node graphs.

## Figures and Tables

**Table 1:** Summary of eight node ranking algorithms involved in this paper and their theoretical foundations.

Algorithm	Type	Core Basis
PageRank	Global Random Walk	Important nodes are voted by significant neighbors, and scores converge iteratively [14].
LeaderRank	Parameter-free Random Walk	A ground node is introduced to make the network strongly connected, and after convergence, the resource of the background node is evenly redistributed to the real nodes [24].
ClusterRank	Local Clustering Method	It combines neighbor influence with a clustering coefficient penalty; nodes with lower clustering coefficients are more conducive to spreading [26].
In-degree	First-order Local Method	A larger in-degree indicates that a node can directly influence more in-neighbors or followers [25].
Adjacency Entropy	Adjacency Information Entropy	Entropy is constructed based on adjacency selection probabilities balanced by in-degree and out-degree [27].
BII	Local-Global Iteration	It iteratively balances the local information represented by the node in-degree and the global influence contributed by in-neighbors [28].
NEM	Neighborhood Entropy Method	It measures neighbor influence capability using the degree-distribution entropy of multi-order in- and out-neighborhoods [25].
KSGR	Structural Gravity and Adaptive Enhancement	It integrates reverse diffusion efficiency, reverse k-shell, structural gravity, bridging capability, and structural diversity.

**Table 2:** Statistical characteristics of six real-world directed network datasets.

Datasets	N	M	${\bar{k}}_{in}$	ρ	$S_{SCC}$
DD68	775	2093	2.7010	0.00349	0.0013
DD687	725	2600	3.5860	0.00495	0.0014
p2p-Gnutella04	10,876	39,994	3.6770	0.00034	0.3969
email-Dept1	309	3031	9.8090	0.03185	0.2783
physician_trust	241	1098	4.5560	0.01898	0.3942
tech-pgp	10,680	24,316	2.2770	0.00021	0.0001

**Note:** denotes the number of nodes in the network, denotes the number of directed edges, denotes the average in-degree, denotes network density, and denotes the proportion of nodes in the largest strongly connected component (SCC) relative to all nodes in the network.

**Table 3:** Comprehensive results of the eight algorithms on the six datasets.

Algorithms	Average Normalized AUC	Average Normalized Final Scale	Top-10 Structural Impact Score
PageRank	0.8604	0.8628	0.7395
LeaderRank	0.9201	0.9132	0.6958
ClusterRank	0.9594	0.9437	0.5157
In-degree	0.8945	0.8540	0.5965
Adjacency Entropy	0.3704	0.3356	0.6679
BII	0.9177	0.8826	0.5965
NEM	0.9533	0.9515	0.5000
KSGR	0.9747	0.9871	0.7060

**Table 4:** Normalized LWCC results based on the DD68 dataset (smaller values indicate better performance).

Algorithm	AUC_LWCC	10% Removal	30% Removal	50% Removal
KSGR	0.2755	0.9006	0.4865	0.0271
Adjacency Entropy	0.2885	0.8929	0.5729	0.0490
In-degree	0.2972	0.9006	0.6284	0.0323
BII	0.3046	0.9006	0.6142	0.0323
LeaderRank	0.2994	0.9006	0.4929	0.1329
ClusterRank	0.3146	0.9006	0.6387	0.0452
PageRank	0.3017	0.9006	0.5032	0.2155
NEM	0.3376	0.9006	0.6877	0.1058

**Table 5:** Normalized LWCC results based on the DD687 dataset (smaller values indicate better performance).

Algorithm	AUC_LWCC	10% Removal	30% Removal	50% Removal
KSGR	0.3453	0.9007	0.6759	0.1255
Adjacency Entropy	0.3579	0.8979	0.6938	0.3503
In-degree	0.3595	0.9007	0.6979	0.2703
BII	0.3615	0.9007	0.6979	0.2648
LeaderRank	0.3621	0.9007	0.6731	0.3834
ClusterRank	0.3681	0.9007	0.6979	0.4428
PageRank	0.3603	0.9007	0.6745	0.4138
NEM	0.3678	0.9007	0.6966	0.4414

**Table 6:** Normalized LWCC results based on the p2p-Gnutella04 dataset (smaller values indicate better performance).

Algorithm	AUC_LWCC	10% Removal	30% Removal	50% Removal
KSGR	0.2289	0.8249	0.3557	0.0006
Adjacency Entropy	0.2335	0.8259	0.3740	0.0027
In-degree	0.3151	0.8657	0.5753	0.1855
BII	0.3186	0.8670	0.5764	0.2272
LeaderRank	0.3272	0.8680	0.5903	0.2933
ClusterRank	0.3326	0.8662	0.5987	0.3253
PageRank	0.3278	0.8681	0.5905	0.2894
NEM	0.3357	0.8664	0.6045	0.3510

**Table 7:** Normalized LWCC results based on the email-Dept1 dataset (smaller values indicate better performance).

Algorithm	AUC_LWCC	10% Removal	30% Removal	50% Removal
KSGR	0.2171	0.6832	0.2871	0.1386
Adjacency Entropy	0.2807	0.8020	0.5050	0.1683
In-degree	0.2837	0.7921	0.4752	0.1683
BII	0.2817	0.7921	0.4653	0.1683
LeaderRank	0.2629	0.7723	0.4851	0.1683
ClusterRank	0.2470	0.7426	0.4059	0.1683
PageRank	0.2626	0.7624	0.4554	0.1386
NEM	0.2550	0.7327	0.4356	0.1683

**Table 8:** Normalized LWCC results based on the physician_trust dataset (smaller values indicate better performance).

Algorithm	AUC_LWCC	10% Removal	30% Removal	50% Removal
KSGR	0.3160	0.8120	0.5470	0.3248
Adjacency Entropy	0.3361	0.9231	0.5812	0.2393
In-degree	0.3509	0.9060	0.6496	0.1795
BII	0.3479	0.8889	0.6325	0.2991
LeaderRank	0.3449	0.8632	0.6410	0.3675
ClusterRank	0.3455	0.8974	0.6410	0.3419
PageRank	0.3415	0.8547	0.6410	0.3675
NEM	0.3474	0.8632	0.6496	0.3846

**Table 9:** Normalized LWCC results based on the tech-pgp dataset (smaller values indicate better performance).

Algorithm	AUC_LWCC	10% Removal	30% Removal	50% Removal
KSGR	0.0698	0.2619	0.0025	0.0018
Adjacency Entropy	0.0846	0.3513	0.0277	0.0087
In-degree	0.0777	0.3167	0.0022	0.0007
BII	0.0817	0.3371	0.0062	0.0011
LeaderRank	0.1227	0.4521	0.1017	0.0091
ClusterRank	0.1187	0.4947	0.0157	0.0010
PageRank	0.1318	0.4806	0.1119	0.0193
NEM	0.1303	0.5568	0.0136	0.0031

**Table 10:** Sensitivity results of the adaptive enhancement parameter α.

α	Average Normalized AUC	Average Normalized Maximum Scale
0.00	0.9013	0.9105
0.25	0.9244	0.9276
0.50	0.9440	0.9449
0.75	0.9620	0.9569
1.00	0.9899	0.9896

**Table 11:** Marginal sensitivity results of KSGR under different alpha intervals.

Alpha Interval	Δ AUC	Δ Final Scale	Interpretation
0.00 → 0.25	0.0231	0.0171	Partial enhancement improves branch signal.
0.25 → 0.50	0.0196	0.0173	Performance continues to improve.
0.50 → 0.75	0.0180	0.0120	Positive but smaller marginal gain.
0.75 → 1.00	0.0279	0.0327	Largest additional improvement.
0.00 → 1.00	0.0886	0.0791	Full module gives best robustness.

**Table 12:** Original network-profile thresholds used for adaptive branch selection in KSGR.

Branch	Network-Profile Conditions
Sparse acyclic quota	N ≤ 1200, R ≤ 0.01, W ≥ 0.95, S ≤ 0.01
Fragmented reciprocal	N ≤ 500, R ≥ 0.65, W ≤ 0.50
Compact reciprocal	N ≤ 250, R ≥ 0.45, W ≥ 0.90
Giant SCC	N ≥ 5000, R ≤ 0.01, W ≥ 0.99, 0.25 ≤ S ≤ 0.45
Structural diversity enhanced	(N ≥ 2000∧R < 0.60) or (0.25 ≤ R< 0.60∧W < 0.80)
Core KSGR	Default branch when none of the above conditions is satisfied

**Table 13:** Perturbation settings for evaluating the robustness of branch-selection thresholds.

Threshold Group	Perturbation Levels
Network size N	0.8N, 0.9N, N, 1.1N, 1.2N
Reciprocity R	0.8R, 0.9R, R, 1.1R, 1.2R
LWCC ratio W	0.8W, 0.9W, W, 1.1W, 1.2W
SCC ratio S	0.8S, 0.9S, S, 1.1S, 1.2S

**Table 14:** Correspondence between Equation (19) components and ablation variants.

Variant	Removed Component
*w*/*o* KS	reverse k-shell
*w*/*o* Reach	reverse local reachability
*w*/*o* Gravity	reverse structural gravity
*w*/*o* Bridge	bridging capability
*w*/*o* HA	hierarchical asymmetry
*w*/*o* DE	reverse diffusion efficiency

**Figure A1:**
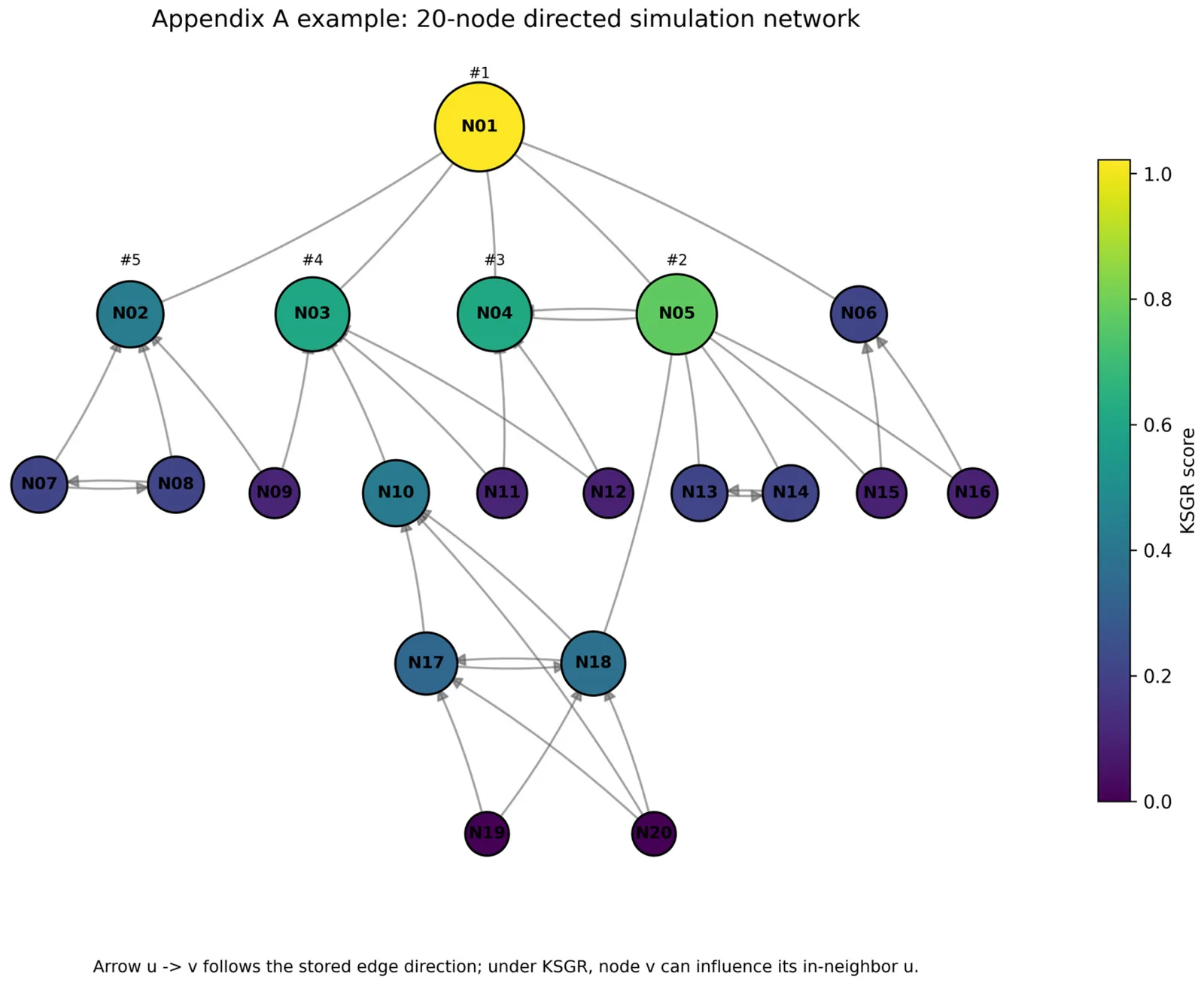
A 20-node directed simulation network used for node-level interpretation. The node size and color are proportional to the KSGR score. The labels above the largest nodes indicate their ranking positions.

## Data Availability

The source code of the KSGR algorithm is openly available in Science Data Bank at https://doi.org/10.57760/sciencedb.37525.

## Appendix A. Interpretable Demonstration of KSGR Based on a Small Directed Simulation Network

### Appendix A.1. Construction of the Simulation Network

To make the ranking mechanism easier to understand, this appendix constructs a 20-node directed simulation network. The edge direction follows the same spreading convention as the main text: if a directed edge u -> v exists, then node v can influence node u along the reverse edge. Thus, nodes with many in-neighbors and broad second-order reverse neighborhoods are expected to obtain higher KSGR values. The simulated network contains 20 nodes and 36 directed edges, with a directed reciprocity of 0.2222, a largest weakly connected component fraction of 1.0000, and a largest strongly connected component fraction of 0.1000. According to the uploaded KSGR implementation, this network is assigned to the core_ksgr branch.

**Table A1:** Directed edge list of the 20-node simulation network.

No.	Directed Edge	No.	Directed Edge	No.	Directed Edge
1	N02 -> N01	13	N14 -> N05	25	N20 -> N10
2	N03 -> N01	14	N15 -> N06	26	N08 -> N07
3	N04 -> N01	15	N16 -> N06	27	N07 -> N08
4	N05 -> N01	16	N17 -> N10	28	N14 -> N13
5	N06 -> N01	17	N18 -> N10	29	N13 -> N14
6	N07 -> N02	18	N19 -> N17	30	N18 -> N17
7	N08 -> N02	19	N20 -> N17	31	N17 -> N18
8	N09 -> N03	20	N19 -> N18	32	N05 -> N04
9	N10 -> N03	21	N20 -> N18	33	N04 -> N05
10	N11 -> N04	22	N12 -> N03	34	N09 -> N02
11	N12 -> N04	23	N15 -> N05	35	N11 -> N03
12	N13 -> N05	24	N18 -> N05	36	N16 -> N05

### Appendix A.2. Node-Level KSGR Values and Interpretation

The KSGR score is a relative structural importance score; a larger value means stronger estimated spreading potential under the reverse spreading convention. In the following tables, S_KSGR denotes the final KSGR score, k_in and k_out denote in-degree and out-degree, KS_R denotes the reverse k-shell index, RLR denotes reverse local reachability, Gravity denotes reverse structural gravity, Bridge denotes the bridging score on the undirected projection, HA denotes hierarchical asymmetry, and RDE denotes reverse diffusion efficiency.

**Table A2:** Main KSGR ranking results and local structural values of all 20 nodes.

Rank	Node	S_KSGR	k_in	k_out	KS_R	RLR
1	N01	1.0221	5	0	1	10.5000
2	N05	0.7709	6	2	1	8.5000
3	N04	0.6071	3	2	1	5.5000
4	N03	0.6068	4	1	1	5.5000
5	N02	0.4221	3	1	1	3.0000
6	N10	0.4190	3	1	1	3.5000
7	N18	0.3749	3	3	1	3.0000
8	N17	0.3327	3	2	1	3.0000
9	N06	0.2115	2	1	0	2.0000
10	N07	0.2113	1	2	1	1.0000
11	N08	0.2113	1	2	1	1.0000
12	N13	0.2093	1	2	1	1.0000
13	N14	0.2093	1	2	1	1.0000
14	N09	0.1018	0	2	0	0.0000
15	N11	0.1006	0	2	0	0.0000
16	N12	0.0952	0	2	0	0.0000
17	N15	0.0952	0	2	0	0.0000
18	N16	0.0952	0	2	0	0.0000
19	N19	0.0017	0	2	0	0.0000
20	N20	0.0000	0	3	0	0.0000

**Table A3:** Complementary KSGR feature values of all 20 nodes.

Node	Gravity	Bridge	HA	RDE
N01	1378.0194	1.6126	2.5257	1.0415
N05	593.0111	1.8814	1.4271	0.5390
N04	291.9222	1.3412	1.1527	0.8109
N03	164.1611	1.7918	1.4469	0.4700
N02	66.0000	1.3412	1.0986	0.0000
N10	146.9500	0.8047	1.0296	0.2231
N18	68.0000	1.0751	0.5465	0.0000
N17	68.0000	0.5365	0.6931	0.0000
N06	6.0000	1.3863	0.9163	0.0000
N07	16.0000	0.0000	0.4520	0.0000
N08	16.0000	0.0000	0.4520	0.0000
N13	16.0000	0.0000	0.4055	0.0000
N14	16.0000	0.0000	0.4055	0.0000
N09	0.0000	1.0986	0.2513	0.0000
N11	0.0000	1.0986	0.2231	0.0000
N12	0.0000	1.0986	0.2231	0.0000
N15	0.0000	1.0986	0.2231	0.0000
N16	0.0000	1.0986	0.2231	0.0000
N19	0.0000	0.0000	0.2231	0.0000
N20	0.0000	0.0000	0.1823	0.0000

The results provide a simple intuitive explanation of the algorithm. Node N01 ranks first because it has five direct in-neighbors and a large second-order reverse coverage range, so it can immediately affect many downstream nodes under reverse spreading. Nodes N05, N04, and N03 form the second tier because they combine relatively high reverse reachability with nontrivial structural gravity. Nodes N10, N18, and N17 do not have the largest direct in-degree, but they obtain moderate scores because they connect the lower-right subnetwork to the upper part of the graph and therefore have visible bridging or frontier roles. Peripheral nodes such as N19 and N20 have no in-neighbors in this example, so their reverse reachability, reverse gravity, and reverse diffusion efficiency are zero, resulting in the lowest KSGR scores. This example shows how KSGR translates the directed local coverage, second-order diffusion potential, backbone position, and bridging information into concrete node-level values.

## Appendix B. Threshold Robustness Analysis of Adaptive Branch Selection

**Table A4:** Detailed perturbed cutoff values for each branch-selection threshold.

***N thresholds***					
Cutoff	−20%	−10%	Original	10%	20%
sparse_n_max	960	1080	1200	1320	1440
fragmented_n_max	400	450	500	550	600
compact_n_max	200	225	250	275	300
giant_n_min	4000	4500	5000	5500	6000
strong_n_min	1600	1800	2000	2200	2400
***R thresholds***					
Cutoff	−20%	−10%	Original	10%	20%
sparse_r_max	0.008	0.009	0.010	0.011	0.012
fragmented_r_min	0.520	0.585	0.650	0.715	0.780
compact_r_min	0.360	0.405	0.450	0.495	0.540
giant_r_max	0.008	0.009	0.010	0.011	0.012
strong_r_hi	0.480	0.540	0.600	0.660	0.720
strong_mid_r_lo	0.200	0.225	0.250	0.275	0.300
strong_mid_r_hi	0.480	0.540	0.600	0.660	0.720
***W thresholds***					
Cutoff	−20%	−10%	Original	10%	20%
sparse_w_min	0.760	0.855	0.950	1.000	1.000
fragmented_w_max	0.400	0.450	0.500	0.550	0.600
compact_w_min	0.720	0.810	0.900	0.990	1.000
giant_w_min	0.792	0.891	0.990	1.000	1.000
strong_mid_w_hi	0.640	0.720	0.800	0.880	0.960
***S thresholds***					
Cutoff	−20%	−10%	Original	10%	20%
sparse_s_max	0.008	0.009	0.010	0.011	0.012
giant_s_min	0.200	0.225	0.250	0.275	0.300
giant_s_max	0.360	0.405	0.450	0.495	0.540
